# Robust Co-clustering to Discover Toxicogenomic Biomarkers and Their Regulatory Doses of Chemical Compounds Using Logistic Probabilistic Hidden Variable Model

**DOI:** 10.3389/fgene.2018.00516

**Published:** 2018-11-01

**Authors:** Mohammad Nazmol Hasan, Md. Masud Rana, Anjuman Ara Begum, Moizur Rahman, Md. Nurul Haque Mollah

**Affiliations:** ^1^Bioinformatics Laboratory, Department of Statistics, University of Rajshahi, Rajshahi, Bangladesh; ^2^Department of Statistics, Bangabandhu Sheikh Mujibur Rahman Agricultural University, Gazipur, Bangladesh; ^3^Department of Veterinary and Animal Sciences, University of Rajshahi, Rajshahi, Bangladesh

**Keywords:** toxicogenomic biomarker, doses of chemical compounds (DCCs), co-clustering, outlying observations, logistic transformation, probabilistic hidden variable model (PHVM), logistic probabilistic hidden variable model (LPHVM)

## Abstract

Detection of biomarker genes and their regulatory doses of chemical compounds (DCCs) is one of the most important tasks in toxicogenomic studies as well as in drug design and development. There is an online computational platform “Toxygates” to identify biomarker genes and their regulatory DCCs by co-clustering approach. Nevertheless, the algorithm of that platform based on hierarchical clustering (HC) does not share gene-DCC two-way information simultaneously during co-clustering between genes and DCCs. Also it is sensitive to outlying observations. Thus, this platform may produce misleading results in some cases. The probabilistic hidden variable model (PHVM) is a more effective co-clustering approach that share two-way information simultaneously, but it is also sensitive to outlying observations. Therefore, in this paper we have proposed logistic probabilistic hidden variable model (LPHVM) for robust co-clustering between genes and DCCs, since gene expression data are often contaminated by outlying observations. We have investigated the performance of the proposed LPHVM co-clustering approach in a comparison with the conventional PHVM and Toxygates co-clustering approaches using simulated and real life TGP gene expression datasets, respectively. Simulation results show that the proposed method improved the performance over the conventional PHVM in presence of outliers; otherwise, it keeps equal performance. In the case of real life TGP data analysis, three DCCs (glibenclamide-low, perhexilline-low, and hexachlorobenzene-medium) for glutathione metabolism pathway dataset as well as two DCCs (acetaminophen-medium and methapyrilene-low) for PPAR signaling pathway dataset were incorrectly co-clustered by the Toxygates online platform, while only one DCC (hexachlorobenzene-low) for glutathione metabolism pathway was incorrectly co-clustered by the proposed LPHVM approach. Our findings from the real data analysis are also supported by the other findings in the literature.

## Introduction

Toxicogenomics studies combines toxicology with several *omics* technologies (genomics, transcriptomics, proteomics, and metabolomics) to assess the risk of toxins (small molecules, peptides, or proteins) and chemical agents (drugs, gasoline, alcohol, pesticides, fuel oil, and cosmetics) in organism (NRC, 2007; Afshari et al., 2011). Through integration of these *omics* technologies with bioinformatics, toxicogenomics can be used to suggest the molecular mechanism of toxicity. This can reduce the cost in terms of time, labor, compound synthesis, and animal use which are main limitations of traditional toxicology work (Nuwaysir et al., 1999; Chen et al., 2012). In drug discovery and development, it is also necessary to assess the doses of chemical compounds (DCCs) toxicity administering these DCCs on individuals for measuring drugs' safety. This assessment can be done by toxicogenomic biomarkers those are upregulated or downregulated by the influence of a set of DCCs on individuals. These toxicogenomic biomarkers can be identified from the extensive gene-treatment expression dataset of target organs of individuals (Fielden et al., 2007; Uehara et al., 2008; Igarashi et al., 2015).

An online toxicogenomic data analysis platform “ToxDB” increases its predictive power based on the pathway level gene expression data (Hardt et al., 2016). It calculates the pathway scores for a chemical compound to identify significant biomarker genes using t-statistic from different pathways. Nevertheless, there is no facility in this platform to study another interesting problem of relationship between gene groups and DCCs groups asserted by Afshari et al. (2011). To address this problem another online platform “Toxygates” produces co-clusters between genes and DCCs using hierarchical clustering (HC) (Nyström-Persson et al., 2017). But HC does not use two-way (gene-DCC) information simultaneously for co-clustering and it is sensitive to outlying observations (García-Escudero et al., 2010). Probabilistic hidden variable model (PHVM) has been developed for co-clustering between words and documents in a text mining problem (Hofmann, 2001). It uses two-way (row-column) information simultaneously during co-clustering. It was also successfully used in detecting hidden patters of biological profiling datasets (Joung et al., 2006; Bicego et al., 2010). Therefore, PHVM would be more effective approach than HC for co-clustering between genes and DCCs which is also supported by Joung et al. (2006). However, the PHVM algorithm is sensitive to outlying observations of gene expression. These outlying observations often occur in the gene expression dataset due to several steps involve in the data generating processes from hybridization to image analysis including scratches or dust on the surface, imperfections in the glass or imperfections in the array production (Gottardo et al., 2006; Upton et al., 2009). The outliers in the dataset may arise following Tukey–Huber contamination model (THCM; Agostinelli et al., 2015) or independent contamination model (ICM; Alqallaf et al., 2009). To overcome the robustness problems of conventional PHVM approach an attempt is made to propose logistic PHVM approach called as LPHVM for robust co-clustering between genes and DCCs to discover toxicogenomic biomarkers and their regulatory DCCs.

## Methods and materials

**Figure 1:**
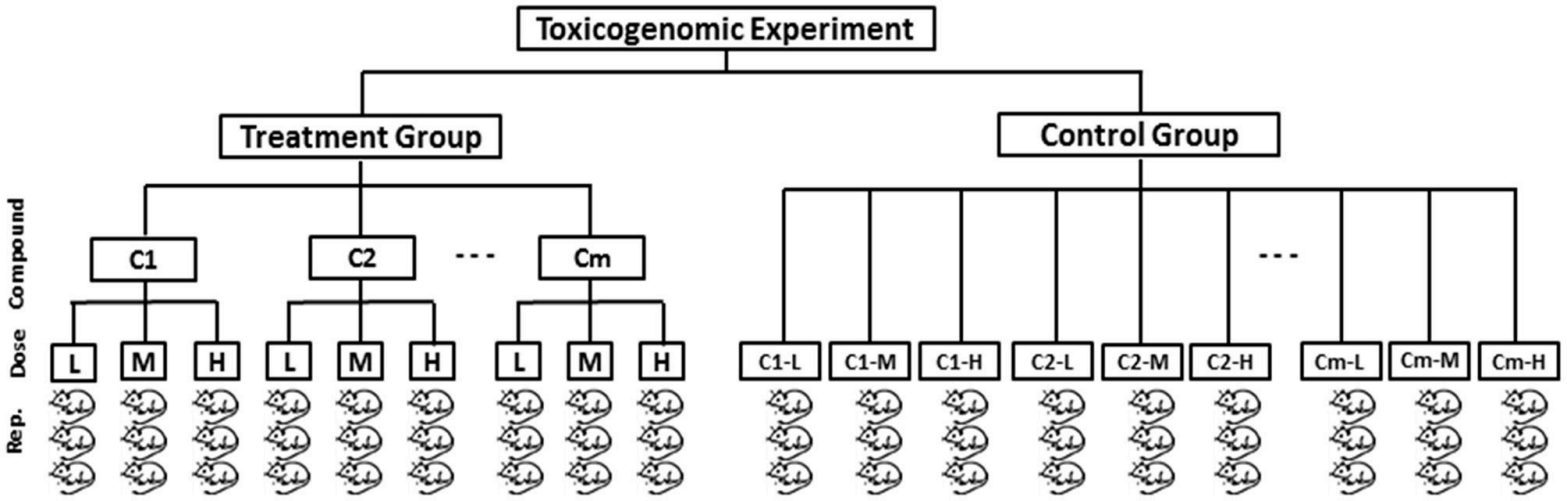
A typical toxicogenomic experimental model for a single time point according to which gene expression data of the animal samples can be collected. In the figure there is a treatment group of animals and a control group of animals from which the fold change gene expression data can be obtained.

Let us consider a toxicogenomic experimental design as described in Figure 1 that reflects Japanese Toxicogenomics Project (TGP) (Uehara, 2010) experiment for a single time point from which the toxygates (Nyström-Persson et al., 2013) data were collected. According to this design, gene expression data of both treatment and control group of animal samples are assumed to be generated. Then the fold change gene expression data for a single time point are computed from the treatment and control group of animals. It can measure the actual treatment (DCCs) effects on the genes. The fold change gene-expression value of a gene is defined as follows:

(1) $$Y_{tlq}=log_{2}\left(\frac{x_{tlq}}{x_{tlq}^{'}}\right)=log_{2}\left(x_{tlq}\right)-log_{2}\left(x_{tlq}^{'}\right),$$

where *Y*_*tlq*_ is the fold change expression value of a gene for the *q*th(*q* = 1, 2, 3) sample under *l*th(l = Low, Middle, High) dose level of the *t*th(*t* = 1, 2, ⋯ , *T*) chemical compound, *x*_*tlq*_ is the expression value of that gene of mentioned sample under the treatment group and $x_{tlq}$ is the expression value of the same gene of the respective control sample. The effect of compound-dose combination or treatment/DCCs on the animal can be measured by ${\bar{Y}}_{tl.}$ which is the average fold change value over the samples. In this paper, our objective is to robust co-clustering between genes and DCCs to discover toxicogenomic biomarkers and their regulatory DCCs from the fold change gene expression data using the proposed LPHVM.

### Logistic transformation of fold change gene expression data

There are two ways to obtain robust estimates in presence of outlying observations (1) applying the robust methods (2) applying conventional methods on the modified dataset. The modification of the outlier contaminated dataset can be done deleting the outlying observations from the dataset or applying transformation on the dataset. Nonetheless, application of robust methods is complicated than using the conventional methods and deletion of outlying observations loses the information of the dataset. Hence, transformation is the better option for reducing outlier effects. Several authors (Box and Cox, 1964; Atkinson, 1982; Carroll, 1982) have been proved that transformation based robust methods outperform the conventional methods in reducing outlier effects. Thus, in this paper we consider logistic transformation for reducing outlier effects from the dataset. Before application of logistic transformation in the dataset we have taken average value $\left({\bar{Y}}_{tl.}\right)$ of the fold change gene expression (*Y*_*tlq*_) over the samples. We denote this average value by *F*(*G*_*i*_, *C*_*j*_) for the convenience of further use. In toxicogenomic data the expression profile of a subset of genes is highly correlated across a subset of conditions/treatments (Madeira and Oliveira, 2004; Bicego et al., 2010; Afshari et al., 2011). Interestingly, in the gene expression or average fold change gene expression data there is a subset of genes which consists an upregulated and a downregulated clusters of genes which is highly correlated over a subset of DCCs. Therefore, we take absolute of the average fold change expression data to merge upregulated and downregulated clusters of genes into a single cluster/subset which are regulated by a subset of DCCs. Thereafter, the subset of genes forms a co-cluster with its regulatory subset of DCCs. Since in this study, we consider all the biomarker and non-biomarker genes (genes are not affected by DCCs) in a pathway, the non-biomarker genes make another co-cluster together with non-regulatory DCCs (which do not affect the expression patterns of the genes in a specific pathway). The term co-cluster refers to the clustering of correlated row (genes) and column (DCCs) simultaneously. Now we apply logistic transformation on the (|*F*(*G*_*i*_, *C*_*j*_)|). If there are extreme values of |*F*(*G*_*i*_, *C*_*j*_)| the logistic transformation bring them within the range of 0–1. The other transformation methods like Box-Cox family of power transformation returns unbounded value for the extreme one. The observed *n*×*m* (gene-DCCs) fold change gene expression data matrix consisting of G = (*G*_1_, *G*_2_, …, *G*_*n*_) genes and C = (*C*_1_, *C*_2_, …, *C*_*m*_) DCCs is transformed using logistic function

$$\#(G_{i},C_{j})=\left(\frac{1}{1+\exp(-\left|F(G_{i},C_{j})\right|}\right)\times 100$$

Similar to other works (Joung et al., 2006; Bicego et al., 2010) we assume the transformed value #(*G*_*i*_, *C*_*j*_) as the count value for applying PHVM.

### Number of co-clusters (k) prediction

As we see from the previous section “logistic transformation of fold change gene expression data” in toxicogenomic dataset there are hidden patters or co-clusters between genes and DCCs. Thus the number of clusters in the DCCs is equal to the number of clusters in the genes. Before applying PHVM it is required to know the number of co-clusters in the dataset. Therefore, in this study, we consider gap statistic (Tibshirani et al., 2001) the most popular and reliable algorithm for predicting the number of co-clusters in the dataset. We use R function “fviz_nbclust” which required packages “factoextra” and “NbClust” (Malika et al., 2014) in order to predict number of co-clusters in the dataset via gap statistic. The detail algorithm of gap statistic is given in the Supplementary Material.

### Robust co-clustering using logistic probabilistic hidden variable model

In order to perform robust co-clustering between genes and DCCs we propose LPHVM approach. We define LPHVM as the application of PHVM on the count valued dataset which is obtained transforming absolute value of the fold change gene expression data by logistic transformation. For this standpoint, let us consider *n*×*m* gene-DCC count valued fold change gene expression data matrix consisting of G = (*G*_1_, *G*_2_, …, *G*_*n*_) genes and C = (*C*_1_, *C*_2_, …, *C*_*m*_) DCCs. LPHVM assumes that there prevail a certain number of unobserved hidden co-clusters or clusters underlying the gene-DCC count valued data matrix. We have estimated the number of co-clusters (*k*) in the dataset using gap statistic algorithm proposed by Tibshirani et al. (2001). Introducing the hidden variable H = (*H*_1_, *H*_2_, …, *H*_*k*_; *r* = 1, 2, …, *k*) the model quantifies the relationships *Pr*(*G*_*i*_|*H*_*r*_), *Pr*(*C*_*j*_|*H*_*r*_), and *Pr*(*G*_*i*_, *C*_*j*_). The following are the probability definition and underlying assumptions of LPHVM accordingly: (1) *Pr*(*H*_*r*_) is the probability of the *r*th co-cluster/cluster and $\overset{k}{\underset{r=1}{\sum }}Pr\left(H_{r}\right)=1$. (2) *Pr*(*G*_*i*_|*H*_*r*_) is the probability of the *i*th gene over the *r*th co-cluster and $\forall H_{r};\;\overset{n}{\underset{i=1}{\sum }}Pr\left(G_{i}|H_{r}\right)=1$. (3) *Pr*(*C*_*j*_|*H*_*r*_) is the probability of the *j*th DCC over the *r*th co-cluster and $\forall H_{r};\;\overset{m}{\underset{j=1}{\sum }}Pr\left(C_{j}|H_{r}\right)=1$. (4) *Pr*(*G*_*i*_, *C*_*j*_) is the joint probability of the *i*th gene and the *j*th DCC and $\overset{n}{\underset{i=1}{\sum }}\overset{m}{\underset{j=1}{\sum }}Pr\left(G_{i},C_{j}\right)=1$. Based on these definition and assumptions we obtain the joint probability of the gene-DCC observed pair (*G*_*i*_, *C*_*j*_) considering hidden co-cluster *H*_*r*_ as follows:

$$Pr\left(G_{i},C_{j}\right)=\;Pr\left(C_{j}\right)Pr(G_{i}|C_{j})$$

Where,

$$Pr\left(G_{i}|C_{j}\right)=\sum_{r\;=\;1}^{k}Pr(G_{i}|H_{r})Pr(H_{r}|C_{j})$$

Applying Bayes' rule, the gene-DCC joint probability *Pr*(*G*_*i*_, *C*_*j*_) can be written as

$$Pr\left(G_{i},C_{j}\right)=\sum_{r\;=\;1}^{k}Pr\left(G_{i}|H_{r}\right)Pr\left(C_{j}|H_{r}\right)Pr(H_{r})$$

So as to estimate the parameters of the model, we need to maximize the total likelihood of the observations:

$$L(G,C)=\sum_{i=1}^{n}\sum_{j\;=\;1}^{m}\#(G_{i},C_{j})logPr(G_{i},C_{j})$$

We have applied the widely used Expectation-Maximization (EM) algorithm (Dempster et al., 1977) for estimating the maximum likelihood parameters of the proposed model. The EM algorithm starts with a random set of initial parameter values and iterates both the expectation (E-step) and maximization (M-step) step alternatively until a certain convergence criteria is satisfied. For this study, we have taken the values of initial parameters from dirichlet distribution and the stopping condition for EM estimation was set to <0.00001 (difference between two log likelihood of successive EM iteration). The E and M-step for the total likelihood can be given as follows:

E-step:

$$Pr\left(H_{r}|G_{i},C_{j}\right)=\;\frac{Pr\left(G_{i}|H_{r}\right)Pr\left(C_{j}|H_{r}\right)Pr(H_{r})}{\sum_{r^{'}}^{k}Pr\left(G_{i}|H_{r^{'}}\right)Pr\left(C_{j}|H_{r^{'}}\right)Pr(H_{r^{'}})}$$

M-step:

$$Pr\left(H_{r}\right)=\;\frac{\sum_{i=1}^{n}\sum_{j=1}^{m}\#(G_{i},C_{j})Pr\left(H_{r}|G_{i},C_{j}\right)}{\sum_{i=1}^{n}\sum_{j=1}^{m}\sum_{r^{'}=1}^{k}\#(G_{i},C_{j})Pr(H_{r^{'}}|G_{i},C_{j})}$$

$$Pr\left(G_{i}|H_{r}\right)=\;\frac{\sum_{j=1}^{m}\#(G_{i},C_{j})Pr\left(H_{r}|G_{i},C_{j}\right)}{\sum_{i^{'}=1}^{n}\sum_{j=1}^{m}\#(G_{i^{'}},C_{j})Pr(H_{r^{'}}|G_{i^{'}},C_{j})}$$

$$Pr\left(C_{j}|H_{r}\right)=\;\frac{\sum_{i=1}^{n}\#(G_{i},C_{j})Pr\left(H_{r}|G_{i},C_{j}\right)}{\sum_{i=1}^{n}\sum_{j^{'}=1}^{m}\#(G_{i},C_{j^{'}})Pr(H_{r^{'}}|G_{i},C_{j^{'}})}$$

Once the parameters *Pr*(*G*_*i*_|*H*_*r*_) and *Pr*(*C*_*j*_|*H*_*r*_) have been estimated the genes and DCCs are clustered independently and co-clustered simultaneously. The gene (*G*_*i*_) and DCC (*C*_*j*_) will belong to co-cluster *r* if

$$\begin{array}{l} Pr\left(G_{i}|H_{r}\right)=argmax_{{}_{r^{\prime }}}Pr(G_{i}|H_{r^{\prime }});i=1,\;2,\\ \;\cdots ,\;n;r=1,2,\;\ldots ,\;k\text{ and}\\ Pr\left(C_{j}|H_{r}\right)=argmax_{{}_{r^{\prime }}}Pr(C_{j}|H_{{}_{r^{\prime }}});j=1,2,\\ \;\ldots ,\;m;r=1,2,\;\ldots ,\;k \end{array}$$

At the same time, if the gene (*G*_*i*_) and the DCC (*C*_*j*_) is grouped into a co-cluster (*r*) and this pair has the highest joint probability *Pr*(*G*_*i*_, *C*_*j*_) in that co-cluster (Figure 3).

### Extraction of toxicogenomic biomarker genes and their regulatory doses of chemical compounds

As described in section “logistic transformation of gene expression data” the biomarker genes form co-clusters with their respective regulatory DCCs. Additionally, the non-biomarker genes in a pathway form another co-cluster with non-regulatory DCCs. The LPHVM grouped the genes and DCCs simultaneously to their respective co-clusters. Zhu et al. (2005) has shown that the PHVM generated co-occurrence probabilities between correlated genes and chemical compounds which co-occur more frequently are higher than others. Biological relationship among these correlated genes and chemical compounds is also stronger. Therefore, we ranked the co-clusters based on the average LPHVM generated joint probability (*Pr*(*G*_*i*_, *C*_*j*_)) of gene-DCC within the co-clusters. The co-cluster having largest average joint probability contains most important biomarker genes and their regulatory DCCs and so on. The non-biomarker genes and non-regulatory DCCs in a dataset of a particular pathway are filtered in a co-cluster by LPHVM which have the smallest average joint probability. Except this co-cluster (co-cluster having smallest average joint probability) others are the co-clusters of biomarker genes and their regulatory DCCs and we define these co-clusters as biomarker co-clusters. We extract the toxicogenomic biomarker genes and their regulatory DCCs from these biomarker co-clusters.

### Up/down-regulated biomarker genes and ranking of doses of chemical compounds

The biomarker co-clusters consisting of biomarker genes and their regulatory DCCs are separated from the whole gene-DCC fold change data matrix which is discussed in the previous section. Within this co-clustering matrix a subset of biomarker genes may be upregulated corresponding to a subset of DCCs or downregulated corresponding to another subset of DCCs. These can be observed from the average fold change value $\left({\bar{Y}}_{tl.}\right)$ of the co-clustering matrix. For example, a biomarker is define as up or down-regulated gene corresponding to the *l*^*th*^ dose level of the *t*^*th*^ chemical compounds if ${\bar{Y}}_{tl.}>0\;$or ${\bar{Y}}_{tl.}<0$. Then this dose of chemical compound is said to be a regulatory DCC. Furthermore, for ranking the biomarker gene regulatory DCCs and their relationships with biomarker genes we have separated a sub matrix of biomarker genes and their regulatory DCCs (biomarker co-clusters) from the LPHVM generated gene-DCC joint probability *Pr*(*G*_*i*_, *C*_*j*_) matrix. The biomarker gene regulatory DCCs are ranked according to their average joint probability value over all biomarkers. We also rank the relationships among biomarker genes and their regulatory DCCs based on their joint probability. The ranking is made considering the formula:

$$\left(\frac{Z_{j/i,j}}{\max\left(Z_{j/i,j}\right)}\right)\times 100$$

where *Z*_*j*_ is the average joint probability of a DCC over the biomarkers or *Z*_*i, j*_ is the joint probability of gene *G*_*i*_ and DCC *C*_*j*_ within the biomarker co-clusters.

### Robustness of the proposed algorithm

We investigate the robustness of the proposed (LPHVM) algorithm and conventional PHVM using simulated datasets in absence and presence of outliers in the dataset based on the co-clustering /clustering error rate (ER). The genes and DCCs which are considered in one co-cluster/cluster in the simulated data are incorrectly assigned in another co-cluster/cluster by the PHVM or LPHVM is considered as the miss co-clustered/clustered observations. The ER is the percentage of miss co-clustered/clustered observations which is calculated as:

$$\left(\frac{tolal\;miss\;co-clustered/clustered\;observations}{Total\;observations}\right)\times 100$$

### Computational steps of LPHVM at a glance

For detecting the toxicogenomic biomarker genes and their regulatory DCCs from the pathway level toxicogenomic dataset using LPHVM the following steps are to be considered for desired outputs:

**Step 1:** Obtain gene expression data of treatment and control group of animals from the toxicogenomic experiment (Figure 1). Thereafter, compute fold change gene expression data using Equation (1) and then make it absolute.

**Step 2:** Apply logistic transformation on the dataset obtain from step 1 and assume the transformed value as count value.

**Step 3:** Estimate the number of co-clusters in the dataset which is obtained from step 2.

**Step 4:** Obtain robust co-clusters applying PHVM on the dataset obtained from step 2 using the number of co-clusters which we get from step 3.

**Step 5:** Calculate average joint probability of gene-DCC within the co-clusters and ranked them.

**Step 6:** Separate the co-clusters of biomarker genes and their regulatory DCCs from the co-cluster which have smallest average joint probability of gene-DCC.

**Step 7:** The genes and DCCs in the separated co-clusters which we get from step 6 are the toxicogenomic biomarkers and their regulatory DCCs.

**Step 8:** A biomarker gene obtains from step 7 may be upregulated corresponding to a DCC or downregulated corresponding to another DCC. A biomarker gene is said to be a up or down-regulated if its average fold change value corresponding to the *l*th dose level of the *t*th chemical compound is ${\bar{Y}}_{tl.}>0\;$or ${\bar{Y}}_{tl.}<0$.

### Simulated datasets

To investigate the performance of the proposed LPHVM algorithm over the conventional PHVM we have simulated two sets of pathway level fold change gene expression data *D*_1_(*n* = 50 × *m* = 30) and *D*_2_(*n* = 50 × *m* = 60) imitating the toxicogenomic experiment given in Figure 1. Alongside these a pathway level dataset considering all time points of toxygates data are analyzed in the real data section. According to this experiment the fold change gene expression data (*Y*_*tlq*_) have been generated using the following model:

**Table:** 

		**DCCs group-1**	**DCCs group-2**	**DCCs group-3**	
	Gene group-11	+*F*11	0	0
	Gene group-12	–*F*12	0	0
*Y*_*tlq*_ =	Gene group-21	0	+*F*21	0	+*N*(0, σ^2^)
	Gene group-22	0	–*F*22	0
	Gene group-3	0	0	0	(2)

In the above model, +*F*11and +*F*21 represent the fold change expression values for upregulated genes under the DCCs group 1 and 2, respectively. Similarly, –*F*12, and –*F*22 represent the fold change expression values for the downregulated genes under the DCCs group 1 and 2, respectively. The 0s represent there is no compound effects on the respective gene group and *N*(0, σ^2^) represents the random error term generated from normal distribution with mean 0 and variance σ^2^. Now if we take absolute value of the fold change gene expression data generated from the above data generating model (2), the fold change gene expression data +*F*11 and –*F*12 will merge into a single gene group-1 and make a co-cluster with their correlated DCCs group-1. Accordingly, +*F*21 and –*F*22 will merge into a single gene group-2 and make a co-cluster with their correlated DCCs group-2. The rest of the genes which are not regulated by any DCCs make a gene group-3 and the DCCs that do not regulate the expression pattern of genes make a DCCs group-3. The gene group-3 and DCCs group-3 together will make another co-cluster. These co-clusters can be retrieved by the LPHVM. In the simulated datasets *n* represents the number of genes (*G*_*i*_; *i* = 1, 2, …, *n*) and *m* represents the number of DCCs (*C*_*j*_; *j* = 1, 2, …, *m*). The data generation procedures for *D*_1_ and *D*_2_ datasets are given in the Supplementary Material.

### Real datasets

Several studies proved that molecular network or pathway based analysis improved the predictive power of gene expression data (Yildirimman et al., 2011; Hofree et al., 2013). Hardt et al. (2016) also analyzed the pathway level data from *in vitro* and *in vivo* experiment of human and rat model. Presently, pathway based analysis in cancer research has also advanced promptly since pathway level analysis able to produce more stable biomarkers (Kim, 2017). Since performance of any method cannot be measured without known dataset. Besides the simulation study, to investigate the performance of the proposed method compare to other existing methods we use two known datasets of glutathione metabolism and PPAR signaling pathways. The fold change expression data of the TGP experiment for glutathione metabolism and PPAR signaling pathway for some selected DCCs of the respective pathway at 24 h time point have been downloaded from toxygates (https://toxygates.nibiohn.go.jp/toxygates/#columns). Because the compounds' toxicity at 24 h time point is more visible compare to other time points (Nyström-Persson et al., 2013). Alongside these a dataset consisting of glutathione metabolism pathway genes and glutathione depleting and non-glutathione depleting compounds (Nyström-Persson et al., 2013) for all time points is also considered for analysis to know about the toxicity of DCCs in other time points.

## Results

### Simulation study

**Figure 2:**
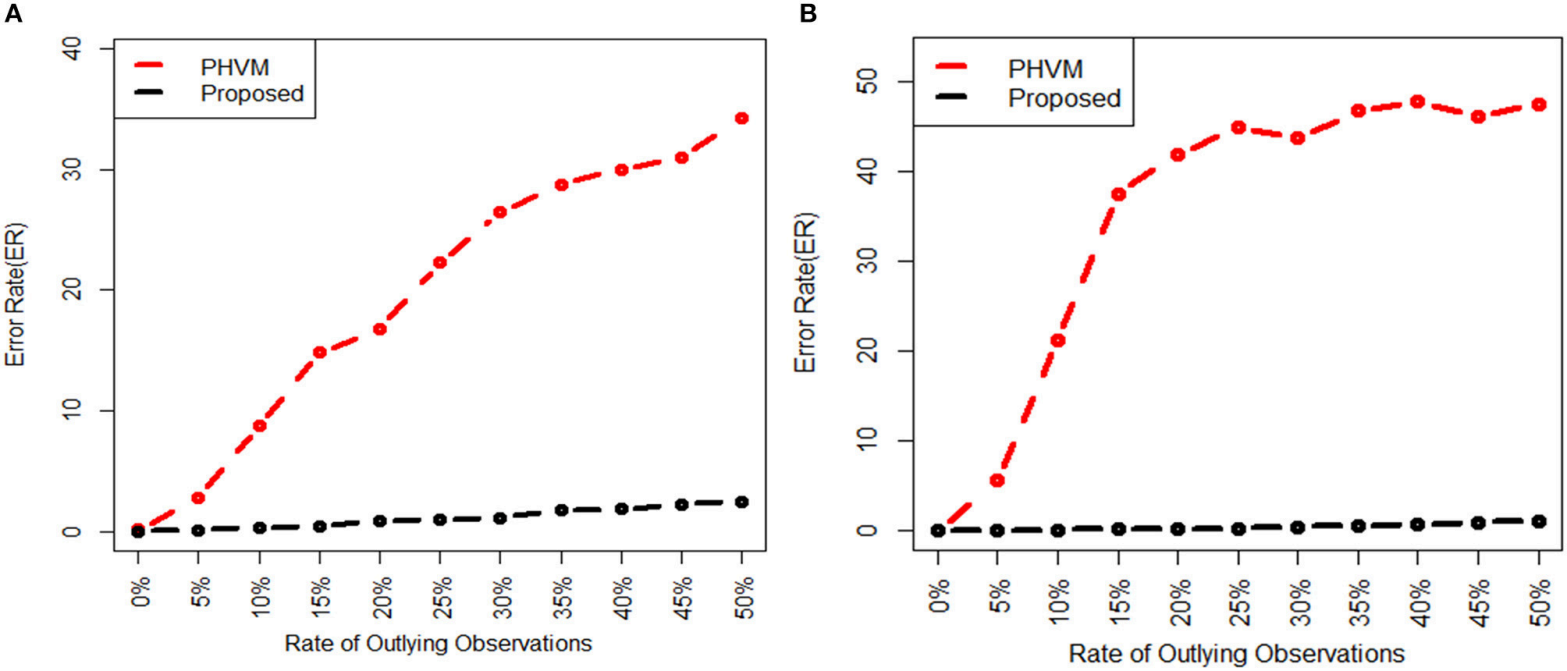
Average gene and doses of chemical compounds co-clustering ER are plotted against the rate of outliers, when each of the data sets are simulated 100 times and outliers in the datasets are introduced using THCM. In the figure **(A)** for *D*_1_ dataset and **(B)** for *D*_2_ dataset.

We investigate the performance of our proposed method (LPHVM) by comparing it with the conventional PHVM using simulated datasets *D*_1_ and *D*_2_ in absence and presence of outlying observations for robust co-clustering between genes and DCCs to discover biomarker genes and their regulatory DCCs. The number of co-clusters/clusters for both of the simulated datasets is estimated as 3 via gap statistic as per the datasets are simulated (Figure S1). For calculating average co-clustering and clustering ER we have simulated each of the datasets 100 times. Every time of data simulation outliers are introduced in the dataset using the data contamination methods THCM and ICM at the same time ER are calculated for PHVM and LPHVM applying these methods on the datasets. The description of the data contamination by outliers, THCM and ICM are given in the Supplementary Material. Here it should be mentioned that in the case of THCM we have contaminated the simulated datasets by 5–50% rate of outliers. Similarly, in the case of ICM we have considered the range of probability of at least one component of the dataset is to be contaminated is 0.14–0.60 for *D*_1_ dataset and 0.165–0.5962 for *D*_2_ dataset. Figure 2 visualizes the average co-clustering ER between genes and DCCs for datasets *D*_1_ and *D*_2_ in absence and presence of outliers when the datasets are contaminated by outliers using the THCM. The Table 1 shows the average co-clustering ER between genes and DCCs in absence and presence of outliers for the simulated datasets *D*_1_ and *D*_2_ when the datasets are contaminated by outliers using ICM. Figure S2 and Table S1 in the Supplementary Material show the average clustering ER for gene and DCCs. It is observed from the mentioned figures and tables that in absence of outlier both of the proposed LPHVM and conventional PHVM approaches produce 0 ER. However, in presence of outlaying observations in the datasets the proposed approach produce far smaller ER than the conventional approach for both of the data contamination methods (THCM and ICM). The simulated data structure, structure of the data when row (gene) and column (DCCs) entities are randomly allocated and proposed method recovered structure of the data are given in the Supplementary Material (Figures S3, S4) for the datasets *D*_1_ and *D*_2_. From these figures it is observed that the proposed algorithm is efficient for co-clustering between genes and DCCs of the pathway level fold change gene expression data. Figure S3C represents the dataset *D*_1_ where all the genes and DCCs are grouped into three co-clusters (co-clusters 1, 2, and 3) and within co-cluster average joint probability of gene-DCC are given in Table 3. From where it is found that co-cluster-1 produces the smallest average joint probability of gene-DCC. Therefore, co-cluster 2 and 3 are the co-cluster of biomarker genes and their regulatory DCCs for the dataset *D*_1_. Similarly, for *D*_2_ dataset co-cluster-3 produces the smallest average joint probability of gene-DCC (Table 3). Thus, co-cluster 1 and 2 are the biomarker co-clusters consisting of biomarker genes and their regulatory DCCs. The biomarker genes and their regulatory DCCs that we get from the biomarker co-clusters of the simulated datasets are given in the Table S9. Ranking of the biomarker regulatory DCCs are performed based on the biomarker gene-DCC joint probability matrix of biomarker co-clusters following the raking method described in sub section (Up/Down-regulated Biomarker Genes and Ranking of Doses of Chemical Compounds). The results are given in the Supplementary Material (Table S10) for both *D*_1_ and *D*_2_ datasets.

**Table 1:** Average values of the gene and doses of chemical compounds co-clustering ER for the simulated datasets *D*_1_ and *D*_2_ when each of the datasets are simulated 100 times and contaminated by outlier using ICM.

**Dataset**	**Method**	**Probability of at least one component in the dataset to be contaminated (ɛ)**						
		**0.00**	**0.14**	**0.26**	**0.36**	**0.45**	**0.53**	**0.60**
*D*_1_	PHVM	0.175	24.675	28.950	32.912	33.500	35.125	38.487
	Proposed	0.025	0.387	0.612	0.725	1.0	1.862	2.500
		**0.00**	**0.165**	**0.3031**	**0.4187**	**0.5154**	**0.5962**
*D*_2_	PHVM	0.00	25.390	26.563	29.554	32.172	39.754
	Proposed	0.00	0.163	0.945	1.481	1.600	2.072

### Analysis of glutathione metabolism pathway data

**Figure 3:**
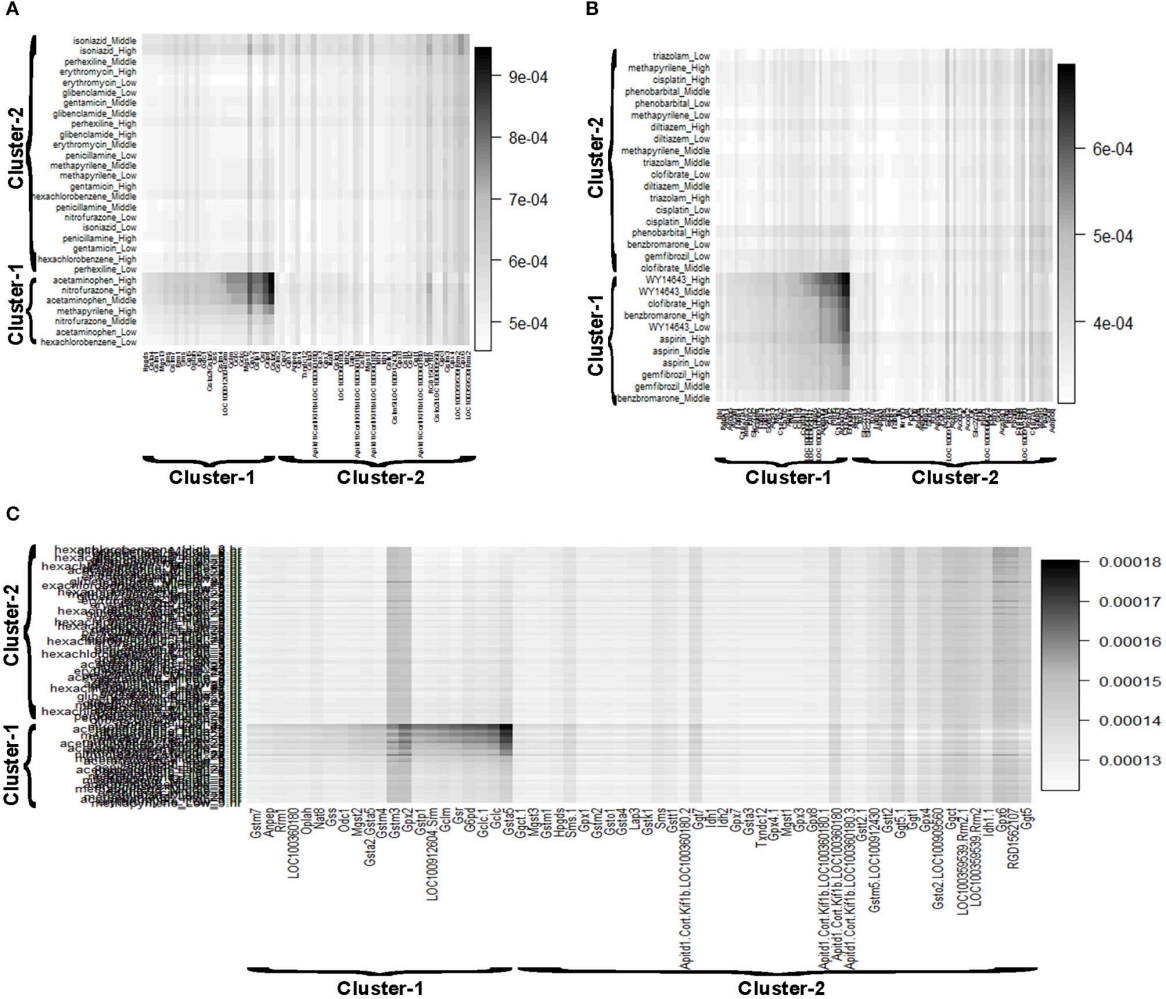
Gene and doses of chemical compounds co-clustered view retrieved from the LPHVM generated gene and DCCs joint probability. **(A)** Represents glutathione metabolism pathway dataset at 24 h time point. **(B)** Represents PPAR signaling pathway dataset at 24 h time point. **(C)** Represents glutathione metabolism pathway dataset for all time points of Toxygates data.

Reactive oxygen species (ROS) are produced by living organisms as a normal product as a result of normal cellular metabolism. However, in presence of environmental pollutants or toxic chemical the production of ROS increased dramatically. It is highly reactive molecules and can damage cell structures such as carbohydrates, nucleic acids, lipids, and proteins and alter their functions. In the liver, glutathione is an important antioxidant; a major detoxification player which scavenges ROS. Thus imbalance in the abundance of ROS and glutathione/antioxidant in favor of ROS in the liver in presence of toxic chemicals/drugs causes' drug induced liver injury. Subsequently, gene expression changes occur simultaneously in response to the glutathione depletion or after the glutathione depletion (Gao et al., 2010; Birben et al., 2012; Nyström-Persson et al., 2013). In order to identify glutathione depletion related biomarker genes and their regulatory DCCs as well as to investigate the performance of the proposed LPHVM approach we use known fold change gene expression dataset of glutathione metabolism pathway. The fold change gene expression dataset consists 62 glutathione metabolism pathway genes, three glutathione depleting compounds (acetaminophen, methapyrilene, and nitrofurazone) and seven non-glutathione depleting compounds (erythromycin, hexachlorobenzene, isoniazid, gentamicin, glibenclamide, penicillamine, and perhexilline) (Nyström-Persson et al., 2013) along with the dose levels (low, middle, and high) for 24 h time point. The number of co-clusters which is required in applying LPHVM for this dataset is estimated as 2 (Figure S1) via gap statistic. Figure 3A shows actual co-clusters in the glutathione metabolism pathway dataset. The genes and DCCs in the co-clusters are given in the Table S2. The average joint probabilities of gene-DCC within the co-clusters are 0.0006196723 and 0.0005331547 (Table 3), respectively for co-cluster-1 and co-cluster-2. Thus, Co-cluster-1 is the co-cluster of biomarker genes and glutathione depleting DCCs as it produces highest average joint probability. The biomarker genes and their regulatory DCCs in co-cluster-1 are given in Table 2. Additionally, the upregulated and downregulated biomarker genes corresponding to their regulatory DCCs are presented in the Figure S7A. For the same dataset the clustering results (heatmap) produced by toxygates are given in Figure S5 where glibenclamide-low, perhexilline-low, and hexachlorobenzene-medium dose level are incorrectly co-clustered whereas only hexachlorobenzene-low dose is incorrectly co-clustered by the proposed LPHVM approach according to Nyström-Persson et al. (2013). The biomarker genes in co-cluster-1 are functionally annotated by the online database DAVID (Huang da et al., 2009) and the results are given in the Tables S5, S6. The results show that the biomarker genes are significant in different biological functions or processes including glutathione metabolism pathway. Ranking of biomarker gene regulatory DCCs and top 20 gene-DCCs relationship along with their ranking score for glutathione metabolism pathway dataset are given in Tables 4, 5. From the tables it is observed that acetaminophen_High, nitrofurazone_High, and acetaminophen_Middle dose etc. are the most important glutathione depleting compounds and Gsta5, G6pd, Gpx2, Gsr, Mgst2, Gstp1, Gclc etc. are the most important biomarker genes. The detail ranked relationships results are given in Table S12. Besides this we have analyzed the same dataset considering all time points (3, 6, 9, and 24 h) by LPHVM to know about toxicity mechanism of the glutathione depleting compounds in other time points. The co-clusters produced by LPHVM are given in Figure 3C. The detail analyzed results of this dataset are given in Tables S4, S11. The proposed LPHVM identified 25 genes for the dataset at 24 h time points and 21 genes for the dataset where all time points are considered as biomarker in the glutathione metabolism pathway among which 18 are common.

**Table 2:** Upregulated and downregulated biomarker genes and their regulatory doses of chemical compounds for real life datasets.

**Dataset**	**Biomarker genes**	**Biomarker gene regulatory DCCs**
Glutathione metabolism pathway	Gsta4, Gstm1, Sms, Rrm1, Odc1, Gsta2/Gsta5, Gss, Gstm4, LOC100912604/Srm, Gclm, Gclc, Mgst2, Gstp1, Gsr, Gpx2, G6pd, Gsta5, Hpgds, Mgst3, Gstm7, Oplah, Ggt5	hexachlorobenzene_Low acetaminophen_Low nitrofurazone_Middle methapyrilene_High acetaminophen_Middle nitrofurazone_High acetaminophen_High
PPAR signaling pathway	Dbi, Acsl1, Acadl, Hmgcs2, Plin2, Slc27a2, Acadm, Fads2, Fabp3, Me1, Sorbs1, Acsl3, Cyp4a2, Aqp7, Cpt1a, Cyp8b1, OC100365047, LOC100910385, Angptl4, Cpt1b, Cpt2, Plin5, Cyp4a3, Acaa1a, Cyp4a1, Ehhadh, Pdpk1, Apoa5, Fabp4, Cyp27a1, Cpt1c, Fabp5	benzbromarone_Middle gemfibrozil_Middle gemfibrozil_High aspirin_Low aspirin_Middle aspirin_High WY14643_Low benzbromarone_High clofibrate_High WY14643_Middle WY14643_High

**Table 3:** Average values of the Gene and DCCs joint probabilities within the co-clusters generated by the proposed LPHVM algorithm for the simulated and real life datasets.

**Dataset**	**Co-cluster-1**	**Co-cluster-2**	**Cocluster-3**
*D*_1_	0.0006095721	0.0010120670	0.0010117088
*D*_2_	0.0005162618	0.0005163485	0.0003147069
Glutathione metabolism pathway	0.0006196723	0.0005331547
PPAR signaling pathway	0.0004471087	0.0003704091

**Table 4:** Biomarker genes regulatory doses of chemical compounds ranking for real datasets (glutathione metabolism and PPAR signaling pathway).

**Dataset**	**Doses of chemical compounds**	**Percent score**
Glutathione metabolism pathway	acetaminophen_High	100.00
	nitrofurazone_High	99.59
	acetaminophen_Middle	95.98
	methapyrilene_High	88.66
	nitrofurazone_Middle	82.24
	acetaminophen_Low	77.84
	hexachlorobenzene_Low	74.57
PPAR signaling pathway	WY14643_High	100.00
	WY14643_Middle	97.59
	clofibrate_High	93.25
	aspirin_High	92.91
	benzbromarone_High	92.25
	WY14643_Low	91.19
	aspirin_Middle	87.93
	aspirin_Low	86.41
	gemfibrozil_High	85.51
	gemfibrozil_Middle	84.52
	benzbromarone_Middle	79.07

**Table 5:** Top 20 (ranked) biomarker gene and their regulatory doses of chemical compound relationships for glutathione metabolism pathway and PPAR signaling pathway datasets.

**Glutathione metabolism pathway**			**PPAR signaling pathway**		
**Chemical compound and dose combination**	**Biomarker gene**	**Ranking score**	**Chemical compound and dose combination**	**Biomarker gene**	**Ranking score**
acetaminophen_High	Gsta5	100.00	WY14643_High	Ehhadh	100.00
nitrofurazone_High	Gsta5	96.26	WY14643_High	Cyp4a1	97.29
acetaminophen_Middle	Gsta5	91.69	WY14643_Middle	Ehhadh	95.32
acetaminophen_High	G6pd	90.85	WY14643_Middle	Cyp4a1	93.17
acetaminophen_High	Gpx2	89.67	WY14643_High	Acaa1a	92.41
nitrofurazone_High	G6pd	89.48	clofibrate_High	Ehhadh	88.93
nitrofurazone_High	Gpx2	89.29	WY14643_Middle	Acaa1a	88.47
acetaminophen_Middle	Gpx2	86.05	clofibrate_High	Cyp4a1	87.34
acetaminophen_Middle	G6pd	85.91	benzbromarone_High	Ehhadh	87.04
acetaminophen_High	Gsr	85.19	WY14643_High	Cyp4a3	86.68
acetaminophen_High	Gstp1	83.54	WY14643_Low	Ehhadh	86.65
nitrofurazone_High	Gsr	83.25	WY14643_High	Plin5	85.99
nitrofurazone_High	Gstp1	81.53	benzbromarone_High	Cyp4a1	85.67
acetaminophen_High	Mgst2	80.46	WY14643_Low	Cyp4a1	85.17
acetaminophen_High	Gclc	80.38	WY14643_High	Cpt2	84.46
methapyrilene_High	Gsta5	80.23	WY14643_High	Cpt1b	84.45
acetaminophen_Middle	Gsr	79.71	WY14643_High	Angptl4	83.99
acetaminophen_High	Gclm	79.56	aspirin_High	Ehhadh	83.60
methapyrilene_High	Gpx2	79.47	WY14643_Middle	Cyp4a3	83.54
nitrofurazone_High	Gclc	78.93	aspirin_High	Cyp4a1	83.10

### Analysis of PPAR signaling pathway data

Peroxisome proliferator-activated receptors (PPARs) PPAR_∝_, PPAR_β/δ_, and PPAR_γ_ are transcription factors which are activated by ligand/drug. They regulate the expression of target genes in response to endogenous and exogenous ligands/chemicals. The PPAR ligands may produce toxicity via receptor-dependent and/or off-target-mediated mechanism(s) (Peraza et al., 2006). To discover PPARs regulated biomarker genes and their regulatory DCCs as well as to investigate the performance of the proposed LPHVM approach we consider known dataset consisting 88 PPAR signaling pathway genes and PPARs related gene regulatory compounds (WY-14643, clofibrate, gemfibrozil, benzbromarone, and aspirin) (Kiyosawa et al., 2006) and some other randomly selected compounds (cisplatin, diltiazem, methapyrilene, phenobarbital, and triazolam) along with their dose levels low, middle and high. The number of hidden co-clusters for this dataset is 2 estimated via gap statistic (Figure S1). The LPHVM generates co-clusters of the PPAR signaling pathway dataset which is shown in Figure 3B. The average joint probabilities of gene-DCC within co-clusters are 0.0004471087 and 0.0003704091 where co-cluster-1 has the larger value than the co-cluster-2. Therefore, co-cluster-1 is the biomarker co-cluster of biomarker genes and their regulatory DCCs. The non-regulated genes and non-regulatory DCCs consist in co-cluster-2. The detail co-clustering results are given in the Table S3. The biomarker genes and their regulatory DCCs in co-cluster-1 are given in Table 2. Additionally, up/down-regulated biomarker genes corresponding to their regulatory DCCs are depicted in the Figure S7B For the same dataset the toxygates co-clustering result using HC given in Figure S6 which shows that acetaminophen-middle and methapyrilene-low are incorrectly co-clustered whereas our proposed method properly co-cluster the DCCs (Table 2) according to the statement of Kiyosawa et al. (2006). Biomarker genes in co-cluster-1 are functionally annotated via DAVID the results are given in the Tables S7, S8. WY14643-High, WY14643-Middle and clofibrate-High are the top most DCCs for regulating PPARs related biomarker genes for detail see Table 4. Top 20 (ranked) relationships between biomarker genes and their regulatory DCCs are given in Table 5 from where it is observed that Ehhadh, Cyp4a1, Acaa1a, Plin5 etc. are the most important biomarker genes and WY14643_High, clofibrate_High, benzbromarone_High, aspirin_High etc. are their important regulatory DCCs in PPAR signaling pathway. The detail results of these relationships are given in the Table S13.

## Discussion and conclusions

Identification of biomarker genes and their regulatory DCCs is one of the most important tasks in the toxicogenomics studies as well as in drug design and development as mentioned before. In this article, we have proposed a robust co-clustering approach based on logistic probabilistic hidden variable model (LPHVM) to detect important biomarker genes and their regulatory DCCs. The proposed LPHVM approach is robust against outlying gene expressions and more flexible and effective than the application of one-way classical clustering approaches (e.g., k-means, fuzzy, HC, etc.) for co-clustering. The proposed method produces robust results by using the logistic transformation of fold-change gene expression data into the conventional PHVM approach. The logistic transformation reduces unusual/outlying observations into the reasonable space without changing the original hidden patterns of genes and DCCs in the dataset. Thus the proposed LPHVM approach produces robust results.

We investigated the performance of the proposed LPHVM method in a comparison with the traditional PHVM and Toxygates online computational platform using simulated and real life TGP gene expression data, respectively. The simulation results showed that the proposed method improves the performance over the conventional PHVM in presence of outlying observations; otherwise, they perform equally. We also demonstrated the performance of the proposed method in a comparison with the online computational platform “Toxygates” using the real life pathway based fold change gene expression datasets collected from the “Toxygates” database. We observed that three DCCs (glibenclamide-low, perhexilline-low, and hexachlorobenzene-medium) for glutathione metabolism pathway dataset as well as two DCCs (acetaminophen-medium and methapyrilene-low) for PPAR signaling pathway dataset were incorrectly co-clustered by the Toxygates online platform, while only one DCC (hexachlorobenzene-low) for glutathione metabolism pathway was incorrectly co-clustered by the proposed LPHVM approach. Our findings from the real life data analysis are also supported by the other findings in the literature (Kiyosawa et al., 2006; Nyström-Persson et al., 2013). Thus the proposed LPHVM outperform over the classical PHVM and “Toxygates” online coputational platform to detect toxicogenomic biomarkers and their regulatory DCCs.

## Data availability

The demo data and R-code are provided at http://www.bbcba.org/softwares/rCoClust.zip.

## Author contributions

MH and MM worked together to develop the algorithm. MH analyzed the data and drafted the manuscript. MM coordinated and supervised the project. MMR, AB, and MR attended at the meeting regarding this article as well as read and approved the final version of the manuscript.

### Conflict of interest statement

The authors declare that the research was conducted in the absence of any commercial or financial relationships that could be construed as a potential conflict of interest.
