# Supplementary material for: Robust Co-clustering to Discover Toxicogenomic Biomarkers and Their Regulatory Doses of Chemical Compounds Using Logistic Probabilistic Hidden Variable Model
Source: Front Genet. 2018 Nov 1;9:516. doi: 10.3389/fgene.2018.00516 (PMC6225736; doi:10.3389/fgene.2018.00516)
Supplement: Supplementary file 1 [file Data_Sheet_1.pdf]

Supplementary Material

Robust co-clustering to discover toxicogenomic biomarkers and their regulatory doses of chemical compounds using logistic probabilistic hidden variable model

Mohammad Nazmol Hasan[a,c], Md. Masud Rana[a], Anjuman Ara Begum[a], Moizur Rahman[b] and Md. Nurul Haque Mollah[a*]

[a]Bioinformatics Lab., Department of Statistics, University of Rajshahi, Rajshahi-6205, Bangladesh.
[b]Department of Veterinary and Animal Sciences, University of Rajshahi, Rajshahi-6205, Bangladesh.
[c]Department of Statistics, Bangabandhu Sheikh Mujibur Rahman Agricultural University, Gazipur-1706, Bangladesh.

[*]Correspondence:
Corresponding Author: Md. Nurul Haque Mollah
E-mail: mollah.stat.bio@ru.ac.bd, nazmol.sat.bsmrau@gmail.com

Contents

1 Gap Statistic
2 Simulated Dataset (D_1)
3 Simulated Dataset (D_2)
4 Simulated Data Contamination by Outliers
5 Tukey-Huber Contamination Model (THCM)
6 Independent Contamination Model (ICM)
7 Application of LPHVM in the case of multiple time points of toxicogenomic Data

Supplementary Tables

Table S1: Average values of the gene and doses of chemical compounds clustering ER for the simulated datasets D_1 and D_2 when each of the datasets are simulated 100 times and contaminated by outlier using ICM.

Table S2: Gene and doses of chemical compounds of the respective co-clusters of the real dataset glutathione metabolism pathway.

Table S3: Gene and doses of chemical compounds of the respective co-clusters of the real dataset PPAR signaling pathway.

Table S4: Gene and doses of chemical compounds of the respective co-clusters of the real dataset glutathione metabolism pathway considering all time points (3hr, 6hr, 9hr and 24hr).

Table S5: Functional annotation of KEGG pathway on the biomarker genes in co-cluster-1dicovered by the proposed LPHVM algorithm in the case of glutathione metabolism pathway data analysis.

Table S6: Functional GO annotation on the biomarker genes in co-cluster-1 discovered by the proposed LPHVM algorithm in the case of glutathione metabolism pathway data analysis.

Table S7: Functional annotation of KEGG pathway on the biomarker genes in co-cluster-1dicovered by the proposed LPHVM in case of the PPAR signaling pathway.

Table S8: Functional GO annotation on the biomarker genes in co-cluster-1 discovered by the proposed LPHVM algorithm in the case of PPAR signaling pathway data analysis.

Table S9: Partitioning the biomarker genes into upregulated and downregulated biomarker genes for simulated datasets.

Table S10: Biomarker genes regulatory deses of chemical compounds ranking for simulated datasets (D_1 and D_2).

Table S11: Biomarker genes regulatory doses of chemical compounds ranking for glutathione metabolism pathway considering all time points (3hr, 6hr, 9hr and 24hr).

Table S12: Ranking of probabilistic relationships between biomarker genes and their regulatory doses of chemical compounds for glutathione metabolism pathway.

Table S13: Ranking of probabilistic relationships between biomarker genes and their regulatory doses of chemical compounds for PPAR signaling pathway.

Supplementary Figures

Figure S1: Gap statistic versus number of hidden co-cluster (k) graph for predicting optimal number of hidden co-clusters/clusters. **(A)** For D_1 dataset. **(B)** For D_2 dataset. **(C)** For glutathione metabolism pathway dataset. **(D)** For PPAR signaling pathway dataset.

Figure S2: Average genes and doses of chemical compounds clustering ER are plotted against the rate of outliers, when each of the datasets is simulated 100 times and outliers in the dataset are introduced in the dataset by THCM. **(A)** Represents gene clustering ER for D_1 dataset. **(B)** Represents DCCs clustering ER for D_1 dataset. **(C)** Represents gene clustering ER for D_2 dataset. **(D)** DCCs clustering ER for D_2 dataset.

Figure S3: View of simulated and proposed method reconstructed data structure for the dataset D_1. **(A)** Represents the original structure of the simulated data. **(B)** Represents the data structure when gene (row entity) and doses of chemical compounds (column entity) randomly allocated. **(C)** Represents the proposed method reconstructed data structure.

Figure S4: View of simulated and proposed method reconstructed data structure for the dataset D_2. **(A)** Represents the original structure of the simulated data. **(B)** Represents the data structure when gene (row entity) and doses of chemical compounds (column entity) are randomly allocated. **(C)** Represents the proposed method reconstructed data structure.

Figure S5: Hirarachical clustering (heatmap) obtained from online toxicogenomic data analysis tool Toxygates for glutathione metabolism pathway genes and some selected compounds (acetaminohen, erythromycin, hexachlorobenzene, isoniazid, gentamicin, glibenclamide, methapyrilene, nitrofurazone, penicillamine and perhexilline) along with dose levels (low, middle, high) at 24 hour time points.

Figure S6: Hirarachical clustering (heatmap) obtained from online toxicogenomic data analysis tool Toxygates for PPAR signaling pathway genes and some selected compounds (acetaminophen, benzbromarone, cisplatin, clofibrate, diltiazem, gemfibrozil, methapyrilene, phenobarbital, triazolam and WY.14643) along with dose levels (low, middle, high) at 24 hour time points.

Figure S7: Upregulated and downregulated biomarker genes corresponding to their regulatory doses of chemical compounds at 24 hour time points. **(A)** For glutathione metabolism pathway dataset. **(B)** For PPAR signaling pathway dataset.

1 Gap Statistic

Suppose, the gene-DCCs count valued fold change gene expression data matrix $\left(G_i, C_j\right)$, $i = 1,2,\ldots,n, j = 1,2,\ldots,m$ consisting of n genes and m DCCs are grouped into k clusters $(U_1, U_2, \ldots, U_k)$ and U_r symbolizes the indices of genes in cluster r, and $n_r = |U_r|$. Now define W_r the sum of pairwise distances for all genes in cluster r

$$W_r = \sum_{i,i' \in U_r} d_{ii'}$$

Where $d_{ii'} = \sum_j \left((G_i, C_j) - (G_{i'}, C_j)\right)^2$ is the squared euclidian distance. Then the pooled within cluster sum of squares (V_k) around the cluster means can be set as

$$V_k = \sum_{r=1}^{k} \left(\frac{1}{2n_r}\right) W_r$$

Similarly, suppose for a^{th}, $a = 1,2,\ldots,A$ reference dataset generated from uniform preparation (Tibshirani *et al*. 2001) are grouped into k clusters then the above parameters can be compute as

$$W_{ar} = \sum_{i,i' \in Ua_r} d_{ii'}$$

$$V_{ak} = \sum_{r=1}^{k} \left(\frac{1}{2n_r}\right) W_{ar}$$

Where W_{ar} is the sum of pairwise distances for all points in cluster r of the ath reference dataset and V_{ak} is the pooled within cluster sum of squares around the cluster means of the ath reference dataset.

The gap statistic "$SapSt(k)$" for the number of clusters k $(k = 1, 2, \ldots, K)$ in the gene-treatment count valued fold change gene expression data matrix can be computed as

$$SapSt(k) = \frac{1}{A} \sum_{a=1}^{A} logV_{ak} - logV_k$$

Now if we let $\bar{y} = \frac{1}{A} \sum_{a=1}^{A} logV_{ak}$, then the standard deviation $s_k = \sqrt{\frac{1}{A} \sum_{a=1}^{A} (logV_{ak} - \bar{y})^2}$ and define $S_k = s_k \sqrt{1 + \frac{1}{A}}$. Finally the optimal number clusters $\hat{k}$ in the data set can be chosen via the smallest k such that $SapSt(k) \geq SapSt(k+1) - S_{k+1}$.

2 Simulated Dataset (D_1)

In the simulated dataset D_1 we have generated fold change gene expression data set of 50 genes for 10 compounds each having 3 dose levels (Low, Middle and High) in the following way. There are three animal samples (replications) for each doses of chemical compound (DCC) and there also three concurrent animal samples for the control group. The compounds and dose levels together with make 30 doses of chemical compounds (DCCs) (C1_low, C1_Middle, …, C10_Middle, C10_High). We have grouped the DCCs into three clusters according to their regulatory mechanism on the pathway level genes. DCCs group-1 (C1_High-C5_High and C1_Middle-C5_Middle) is upregulate the gene group-11 ($G1 - G7$) and downregulate the gene group-12 ($G8 - G10$). The fold change values (+$F$11) for the DCCs (C1_High-C5_High) and (C1_Middle-C5_Middle) in the DCCs group-1 are +3.00 and +2.50 respectively for gene group-11 according to the data generating model (2) given in section simulated datasets of the main paper. The fold change values (-$F$12) for the DCCs (C1_High-C5_High) and (C1_Middle-C5_Middle) in the DCCs group-1 are -3.00 and -2.50 respectively for the gene group-12. Similarly, for the DCCs (C6_High-C10_High) and (C6_Middle-C10_Middle) in the DCCs group-2 the fold change values (+$F$21 and -$F$22) for the gene group-21 (G11-G17) and 22 (G18-G20) are (+3.00 and +2.50) and (-3.00 and -2.50) respectively. The rest of the inputs in the simulated data matrix D_1 are 0s. An error term $N(0,0.35)$ is added with each of the input of the matrix D_1. After that, if we take the absolute of the dataset D_1 gene group-11 and 12 are merged into a single cluster and make a co-cluster-1 with DCCs group-1. Similarly, gene group -21 and 22 are merged into a single cluster and make a co-cluster-2 with DCCs group-2. The rest of the genes (G21-G50) make a co-cluster-3 with the rest DCCs of the data matrix D_1.

3 Simulated Dataset (D_2)

In the simulated dataset D_2 we have generated fold change gene expression data set of 50 genes for 20 compounds. The dose levels for each compound and replications are similar to dataset D_1. The compounds in combination with dose levels together with make 60 (C1_low, C1_Middle, …, C20_Middle, C20_High) DCCs. These DCCs are grouped into three clusters according to their regulatory capacity on the pathway level genes. DCCs group-1 (C1_High-C7_High and C1_Middle-C7_Middle) is upregulate the gene group-11 ($G1 - G7$) downregulate the gene group-12 ($G8 - G10$). The fold change values (+$F$11) for the DCCs (C1_High-C7_High) and (C1_Middle-C7_Middle) in the DCCs group-1 are +3.00 and +2.50 respectively for the gene group-11 according to the data generating model (2). The fold change values (-$F$21) for the DCCs (C1_High-C7_High) and (C1_Middle-C7_Middle) in the DCCs group-1 are -3.00 and -2.50 respectively for the gene group-12. Likewise, for the DCCs (C11_High-C17_High) and (C11_Middle-C17_Middle) in the DCCs group-2 the fold change values (+$F$21 and –$F$22) for the gene group-21 (G11-G17) and 22 (G18-G20) are (+3.00 and +2.50) and (-3.00 and -2.50) respectively. The rest of the inputs in the simulated data matrix D_2 are 0s. An error term $N(0,0.35)$ is added with each of the input of the matrix D_2. After that, if we take the absolute of the dataset D_2 gene group-11 and 12 are merged into a single cluster and make a co-cluster-1 with DCCs group-1. Similarly, gene group -21 and 22 are merged into a single cluster and make a co-cluster-2 with DCCs group-2. The rest of the genes (G21-G50) make a co-cluster-3 with the rest DCCs of the data matrix D_2.

4 Simulated Data Contamination by Outliers

Outlier is an observation in the dataset arises for some unexpected circumstances which deviate from the actual value of the observation. It is a common problem for gene expression/fold change gene expression data analysis. To compare the performance of the proposed method with conventional PHVM in absence or presence of outlying observations in the data, we contaminate the fold change gene expression data inputting outliers in the simulated datasets. We define outlying values in the simulated data which are five times larger than the maximum value of original simulated data. These outliers may arise in the dataset casewise following the Tukey-Huber contamination model (THCM) (Tukey, 1962; Huber, 1964) or independent cellwise following the independent contamination model (ICM) (Alqallaf et al., 2009). In our gene-DCCs fold change gene expression toxicogenomic data matrix each gene in the row is a case and each input in the matrix is a cell. Therefore, for investigating the robustness of the proposed (LPHVM) algorithm over the conventional (PHVM) algorithm we have contaminated the simulated datasets by outliers casewise and independent cellwise. The descriptions of casewise or THCM and independent cellwise or ICM are given in the supplementary material.

5 Tukey-Huber Contamination Model (THCM)

We have generated two datasets D_1 (30×50) and D_2 (60×50) following the simulated data generation model (2); the numbers within parenthesis represent the dimension of the datasets. To examine the robustness of the proposed method we have contaminated the simulated gene-DCCs fold change gene expression datasets genewise/casewise using the following the THCM (Tukey, 1962; Huber, 1964):

$$Z(D_d) = (1-\varepsilon)Z_0(D_d) + \varepsilon\tilde{Z}(D_d);\ 0 \le \varepsilon \le 0.5 \text{ and } d = 1, 2$$

Where, ε is the small proportion of cases (genes) to be contaminated, $\tilde{Z}$ is the distribution of the expression values of the outlier contaminated genes, Z_0 is the nominal distribution by which we have generated the simulated data and D_d is the simulated data matrix D_1 or D_2. In this study, to measure the robustness of the proposed algorithm we have considered wide range values of ε (5%-50%).

6 Independent Contamination Model (ICM)

We also have contaminated the simulated gene-DCCs fold change gene expression data matrixes using the following the ICM (Alqallaf *et al*,. 2009):

$$D_r = (I - B_\varepsilon)D_{d(0)} + B_\varepsilon\widetilde{D_d}\ ;\ d = 1,2$$

Where $D_{d(0)} \sim Z_0$, $\widetilde{D_d} \sim \tilde{Z}$, I is a $(m \times m)$ identity matrix, $B_\varepsilon = diag(B_1, B_2, \cdots, B_m)$and B_j are independent $Birn(1, \varepsilon)$ it indicates that ε is the probability of each component in D_d to be contaminated. Additionally, the probability $\bar{\varepsilon}$ indicates that at least one component in D_d to be contaminated. Where $\bar{\varepsilon} = 1 - (1 - \varepsilon)$. For dataset D_1 and D_2 we have considered the value of $\bar{\varepsilon}$ (0.1396, 0.2603, 0.3645, 0.4545, 0.5321 and 0.5990) and (0.1650, 0.3031, 0.4187, 0.5154 and 0.5962) respectively.

7 Application of LPHVM in the case of multiple time points of toxicogenomic Data

The toxygates data were collected from The Japanese Toxicogenomics Project (TGP) (Uehara et al., 2010). The project generated large scale gene expression data with a view to measure the effect of chemical compounds on liver and kidney as primary target organs in both *in vivo* and *in vitro* experiments. The *in vivo* experiments were set out on *Rattus Norvegicus*, in combination with compounds each with three dose levels (low, middle and high), four time points (3 hour, 6 hour, 9 hour and 24 hour) and two organs (liver and kidney). Besides each of the compound, dose and time combination treatment group of animal, a control animal was also available in the experiment. The fold change gene expression data can be computed from the gene expression data of the treatment and control group of animals produced by the TGP experiment. In the toxicogenomic data there are subsets of genes which expression profile are highly associated with the subsets of compound-dose-time combinations. Say, ($C_1D_HT_4$, $C_5D_MT_2$, $C_1D_MT_4$, $C_5D_HT_2$ … $C_{11}D_HT_3$) a group of compound-dose-time combinations regulates or associated with the expression pattern of a gene group (G_5, G_9, G_1, … G_{30}). Similarly, another group of compound-dose-time combinations ($C_8D_HT_4$, C_4 D_MT_2, $C_6D_HT_4$, $C_{12}D_HT_2$ … $C_{15}D_MT_3$) regulates the expression patters of another group (G_4, G_8, G_{11}, … G_{20}) of genes and so on. In the regulated group of genes there are an upregulated subset and a downregulated subset of genes. Similarly, if we consider Toxygates data at single time point, the subsets of compound-dose combinations or doses of chemical compounds (DCCs) will we correlated or associated with the subsets of genes. For example, the subset of DCCs (C_1D_H, C_5D_M, C_1D_M, C_5D_H … $C_{11}D_H$) regulates or associated with the expression pattern of a subset of genes (G_3, G_{10}, G_1, … G_{30}). Similarly, another subset of compound-dose-time combinations (C_8D_H, C_4D_M, C_6D_H, $C_{12}D_H$ … $C_{15}D_M$) regulates the expression patters of another subset (G_9, G_8, G_{15}, … G_{20}) of genes and so on. Since PHVM can be applied for co-clustering the genes and treatments/conditions. Therefore, our proposed algorithm LPHVM is efficient to retrieve these hidden structures in the toxicogenomic dataset by co-clustering the correlated genes treatment combinations for single or any multiple of time points.

Table S1: Average values of the gene and doses of chemical compounds clustering ER for the simulated datasets D_1 and D_2 when each of the datasets are simulated 100 times and contaminated by outlier using ICM.

Dataset	Method	Probability of at least one component in the dataset to be contaminated ($\bar{\mathring{a}}$)						
		**0.00**	**0.14**	**0.26**	**0.36**	**0.45**	**0.53**	**0.60**
$\boldsymbol{D_1}$	**Gene Clustering**							
	PHVM	0.280	25.080	30.320	34.940	35.580	38.360	40.500
	Proposed	0.040	0.620	0.980	1.120	1.520	2.300	3.180
	**Doses of chemical compounds clustering**							
	PHVM	0.00	24.000	26.666	29.866	31.366	34.400	36.466
	Proposed	0.00	0.000	0.000	0.066	0.853	1.133	1.580
$\boldsymbol{D_2}$		**0.00**	**0.165**	**0.3031**	**0.4187**	**0.5154**	**0.5962**	
	**Gene Clustering**							
	PHVM	0.00	23.080	25.620	27.620	30.540	37.820	
	Proposed	0.00	0.340	1.060	1.520	1.760	2.500	
	**Doses of chemical compounds clustering**							
	PHVM	0.00	27.316	30.683	32.500	37.366	40.200	
	Proposed	0.00	0.116	0.966	1.450	1.466	2.350	

Table S2: Gene and doses of chemical compounds of the respective co-clusters of the real dataset glutathione metabolism pathway.

Co-cluster-1		Co-cluster-2	
**Gene**	**Doses of chemical compounds**	**Gene**	**Doses of chemical compounds**
Hpgds, Gsta4, Gstm1, Mgst3, Sms, Gstm7, Sms, Rrm1, Oplah, Ggt5, Odc1, Gsta2/Gsta5, Gss, Gstm4, LOC10091260 4-/Srm, Gclm, Gclc, Mgst2, Gstp1, Gsr, Gpx2, G6pd, Gsta5	hexachlorobenzene_Low acetaminophen_Low nitrofurazone_Middle methapyrilene_High acetaminophen_Middle nitrofurazone_High acetaminophen_High	Gstm2, Ggct, Anpep, Gpx1, Gpx4, Apitd1/Cort/Kif1b/L-OC100360180, Txndc12, Gpx7, Gsta3, Gpx3, Nat8, LOC100360180, Gsto1, dh2, Lap3, Apitd1/Cort/Kif1b/L-OC10036-0180, Gstt2, Idh1, Apitd1/Cort/Kif1b/LOC100 -360180, Mgst1, Idh1, Gstk1, Gstm5/LOC100912430, Gpx8, Gstt1, Gstt2, Ggt1, Apitd1/Cort/Kif1b/LOC100 -360180, RGD1562107, Ggt7, Gsto2/LOC1009095-60, Gstm3, Ggct, Gpx4, Gpx6, LOC100359539/Rrm2, LOC100359539/Rrm2	perhexiline_Low hexachlorobenzene_High gentamicin_Low penicillamine_High isoniazid_Low nitrofurazone_Low penicillamine_Middle hexachlorobenzene_Middle gentamicin_High methapyrilene_Low methapyrilene_Middle penicillamine_Low erythromycin_Middle glibenclamide_High perhexiline_High glibenclamide_Middle gentamicin_Middle glibenclamide_Low isoniazid_High erythromycin_Low erythromycin_High perhexiline_Middle isoniazid_Middle

Table S1 shows co-cluster members (genes and DCCs) of the glutathione metabolism pathway dataset. The co-cluster-1 produces the larger average joint probability value compare to the co-cluster-2. Thus, co-cluster-1 is the co-cluster of biomarker genes and their regulatory DCCs for the glutathione metabolism pathway dataset.

Table S3: Gene and doses of chemical compounds of the respective co-clusters of the real dataset PPAR signaling pathway.

Co-cluster-1		Co-cluster-2	
**Gene**	**Doses of chemical compounds**	**Gene**	**Doses of chemical compounds**
Dbi, Pdpk1, Acsl1, Apoa5, Fabp4, Acadl, Cyp27a1, Hmgcs2, Plin2, Slc27a2, Acadm, Fads2, Fabp3, Me1, Sorbs1, Acsl3, Cyp4a2, Cpt1c, Aqp7, Cpt1a, Cpt1a, Cyp8b1, LOC10036-5047 LOC100365047, Fabp5, LOC100910-385, Angptl4, Cpt1b, Cpt2, Plin5, Cyp4a3, Acaa1a, Cyp4a1, Ehhadh	benzbromarone_Middle gemfibrozil_Middle gemfibrozil_High aspirin_Low aspirin_Middle aspirin_High WY14643_Low benzbromarone_High clofibrate_High WY14643_Middle WY14643_High	Acsl4, Acsl1, Rxra, Slc27a1, Slc27a5, Rxrg, Fabp7, Apoa1, Ubb, Scp2, Scp2, Fabp1, Ilk, Nr1h3, Lpl, Pck2, Pltp, Ppard, Apoc3, Fabp2, Scd2, Rxrg, Apoa2, Pck1, Acsl5, LOC100912469, Acsl5, Olr1, Acox3, Gk, Acox2, Pltp, Slc27a4, Plin1, LOC100909612, Pck2, Plin4, Scd, Acsbg1, Pparg, Rxrb, Ppard, Cyp4a8, Cyp4a8, LOC100912469, Acsl1, Cyp7a1, Fabp6, Acsl6, Ppara, Scd, Adipoq	clofibrate_Middle gemfibrozil_Low benzbromarone_Low phenobarbital_High cisplatin_Middle cisplatin_Low triazolam_High diltiazem_Middle clofibrate_Low triazolam_Middle methapyrilene_Middle diltiazem_Low methapyrilene_Low diltiazem_High phenobarbital_Low phenobarbital_Middle cisplatin_High methapyrilene_High triazolam_Low

Table S2 shows co-cluster members (genes and DCCs) of the PPAR signaling pathway dataset. The co-cluster-1 produces the larger average joint probability value compare to the co-cluster-2. Thus, co-cluster-1 is the co-cluster of biomarker genes and their regulatory DCCs for the PPAR signalling pathway dataset.

Table S4: Gene and doses of chemical compounds of the respective co-clusters of the real dataset glutathione metabolism pathway considering all time points (3hr, 6hr, 9hr and 24hr).

**Co-cluster-1**		**Co-cluster-2**	
**Gene**	**Doses of chemical compounds**	**Gene**	**Doses of chemical compounds**
Gstm7, Anpep, Rrm1, LOC100360180, Oplah, Nat8, Gss, Odc1, Mgst2, Gsta2.Gsta5, Gstm4, Gstm3, Gpx2, Gstp1, LOC100912604.Srm, Gclm, Gsr, G6pd, Gclc.1, Gclc, Gsta5	methapyrilene_Low_3.hr, erythromycin.ethylsuccinate_Low_6.hr acetaminophen_High_3.hr, methapyrilene_Low_24.hr, isoniazid_Middle_9.hr, erythromycin.ethylsuccinate_Low_9.hr, isoniazid_Middle_3.hr, methapyrilene_Middle_3.hr, erythromycin.ethylsuccinate_High_9.hr, acetaminophen_Low_3.hr, perhexiline_Middle_9.hr, nitrofurazone_Middle_3.hr, nitrofurazone_High_3.hr, methapyrilene_High_3.hr, gentamicin_High_6.hr, penicillamine_High_6.hr, acetaminophen_Low_24.hr, penicillamine_High_9.hr, isoniazid_Low_3.hr, isoniazid_High_9.hr, acetaminophen_Low_9.hr, methapyrilene_Middle_6.hr, isoniazid_High_24.hr, nitrofurazone_Middle_24.hr, nitrofurazone_Middle_6.hr, methapyrilene_High_6.hr, acetaminophen_Middle_9.hr, nitrofurazone_Low_9.hr, acetaminophen_Middle_24.hr, nitrofurazone_Middle_9.hr, nitrofurazone_Low_6.hr, methapyrilene_High_9.hr, methapyrilene_High_24.hr, nitrofurazone_High_6.hr, nitrofurazone_High_9.hr, acetaminophen_High_24.hr, nitrofurazone_High_24.hr	Ggct.1, Mgst3, Gstm1, Hpgds, Sms.1, Gpx1, Gstm2, Gsto1, Gsta4, Lap3, Gstk1, Sms, Gstt1, Apitd1.Cort.Kif1b.LOC100360180.2, Ggt7, Idh1, Idh2, Gpx7, Gsta3, Txndc12, Gpx4.1, Mgst1, Gpx3, Gpx8, Apitd1.Cort.Kif1b.LOC100360180.1, Apitd1.Cort.Kif1b.LOC100360180, Apitd1.Cort.Kif1b.LOC100360180.3, Gstt2.1, Gstm5.LOC100912430, Gstt2, Ggt5.1, Ggt1, Gpx4, Gsto2.LOC100909560, Ggct, LOC100359539.Rrm2.1, LOC100359539.Rrm2, Idh1.1, Gpx6, RGD1562107, Ggt5	isoniazid_Low_24.hr, isoniazid_High_3.hr, glibenclamide_High_6.hr, erythromycin.ethylsuccinate_Middle_9.hr, penicillamine_Middle_24.hr, penicillamine_Low_3.hr, hexachlorobenzene_Middle_9.hr, perhexiline_Middle_3.hr, glibenclamide_High_9.hr, methapyrilene_Middle_9.hr, glibenclamide_High_24.hr, erythromycin.ethylsuccinate_High_24.hr, perhexiline_Low_9.hr, glibenclamide_Middle_6.hr, gentamicin_Low_9.hr, gentamicin_High_24.hr, perhexiline_High_24.hr, hexachlorobenzene_Low_24.hr, penicillamine_Low_24.hr, acetaminophen_Low_6.hr, glibenclamide_Low_9.hr, gentamicin_Middle_6.hr, acetaminophen_Middle_3.hr, penicillamine_Middle_9.hr, perhexiline_Low_24.hr, erythromycin.ethylsuccinate_Middle_24.hr, glibenclamide_Low_3.hr, gentamicin_Low_24.hr, acetaminophen_High_9.hr, penicillamine_Low_9.hr erythromycin.ethylsuccinate_High_6.hr, nitrofurazone_Low_3.hr, perhexiline_Low_3.hr, hexachlorobenzene_Middle_3.hr, perhexiline_High_3.hr, penicillamine_High_3.hr, gentamicin_Middle_9.hr, isoniazid_Low_6.hr, erythromycin.ethylsuccinate_High_3.hr, hexachlorobenzene_High_24.hr, penicillamine_High_24.hr, acetaminophen_High_6.hr, perhexiline_High_9.hr, penicillamine_Middle_6.hr, nitrofurazone_Low_24.hr, methapyrilene_Low_6.hr, hexachlorobenzene_Low_6.hr, gentamicin_Low_3.hr, hexachlorobenzene_High_9.hr, gentamicin_High_9.hr, gentamicin_Middle_3.hr, perhexiline_High_6.hr, glibenclamide_Low_24.hr, hexachlorobenzene_Low_3.hr, isoniazid_Middle_6.hr, gentamicin_High_3.hr, erythromycin.ethylsuccinate_Low_24.hr, isoniazid_High_6.hr, erythromycin.ethylsuccinate_Middle_3.hr, isoniazid_Low_9.hr, glibenclamide_Middle_3.hr, methapyrilene_Middle_24.hr, hexachlorobenzene_Low_9.hr, perhexiline_Low_6.hr, erythromycin.ethylsuccinate_Low_3.hr, hexachlorobenzene_Middle_24.hr, isoniazid_Middle_24.hr, glibenclamide_Middle_24.hr, gentamicin_Low_6.hr, erythromycin.ethylsuccinate_Middle_6.hr, penicillamine_Low_6.hr, gentamicin_Middle_24.hr acetaminophen_Middle_6.hr, penicillamine_Middle_3.hr, hexachlorobenzene_Middle_6.hr, perhexiline_Middle_24.hr, methapyrilene_Low_9.hr, glibenclamide_High_3.hr, hexachlorobenzene_High_6.hr, perhexiline_Middle_6.hr, glibenclamide_Low_6.hr, glibenclamide_Middle_9.hr, hexachlorobenzene_High_3.hr

Table S5: Functional annotation of KEGG pathway on the biomarker genes in co-cluster-1dicovered by the proposed LPHVM algorithm in the case of glutathione metabolism pathway data analysis.

**Term**	**Count**	**%**	**P-value**	**FDR**	**Genes**
rno00480:Glutathione metabolism	20	90.91	2.76E-41	2.04E-38	Gsta4, Gstm1, Mgst3, Sms, Gstm7, Rrm1, Oplah, Ggt5, Odc1, Gsta2/Gsta5, Gss, Gstm4, Gclm, Gclc, Mgst2, Gstp1, Gsr, Gpx2, G6pd, Gsta5
rno00980:Metabolism of xenobiotics by cytochrome P450	9	40.91	3.29E-12	2.43E-9	Mgst2, Mgst3, Gstm7, Gsta5, Gsta2/Gsta5, Gstp1, Gstm1, Gstm4, Gsta4
rno00982:Drug metabolism - cytochrome P450	9	40.91	3.70E-12	2.73E-9	Mgst2, Mgst3, Gstm7, Gsta5, Gsta2/Gsta5, Gstp1, Gstm1, Gstm4, Gsta4
rno05204:Chemical carcinogenesis	9	40.91	2.88E-11	2.12E-8	Mgst2, Mgst3, Gstm7, Gsta5, Gsta2/Gsta5, Gstp1, Gstm1, Gstm4, Gsta4
rno01100:Metabolic pathways	9	40.91	0.011	7.81	Rrm1, Ggt5, G6pd, Gclm, Odc1, Gclc, Sms, Gss, Hpgds
rno00590:Arachidonic acid metabolism	3	13.64	0.018	12.54	Ggt5, Gpx2, Hpgds

Table S6: Functional GO annotation on the biomarker genes in co-cluster-1 discovered by the proposed LPHVM algorithm in the case of glutathione metabolism pathway data analysis.

**Term**	**Count**	**%**	**P-value**	**FDR**	**Genes**
GO:0098754~detoxification	7	31.82	5.65E-10	4.16E-7	Mgst2, Mgst3, Gpx2, Gsr, Gstm7, Gstp1, Gstm1
GO:0008152~metabolic process	20	90.91	5.26E-4	0.387	Hpgds, Gsta4, Gstm1, Sms, Gstm7, Rrm1, Oplah, Ggt5, Odc1, Gsta2/Gsta5, Gss, Gstm4, Gclm, Gclc, Mgst2, Gstp1, Gsr, Gpx2, G6pd, Gsta5
GO:0050896~response to stimulus	18	81.81	0.002	1.801	Gsta4, Gstm1, Gstm7, Rrm1, Ggt5, Odc1, Gsta2/Gsta5, Gss, Gstm4, Gclm, Gclc, Mgst2, Gstp1, Gsr, Gpx2, G6pd, Gsta5, Mgst3
GO:0051704~multi-organism process	6	27.27	0.082	46.90	Mgst2, Gpx2, Odc1, Gsr, Gstp1, Hpgds

Table S7: Functional annotation of KEGG pathway on the biomarker genes in co-cluster-1dicovered by the proposed LPHVM in case of the PPAR signaling pathway.

Term	Count	%	P-value	FDR	Genes
rno03320:PPAR signaling pathway	26	81.25	3.44E-50	3.28E-47	Cpt1b, Aqp7, Cpt1c, Pdpk1, Cpt1a, Cyp27a1, Fabp3, Ehhadh, Acaa1a, Acadm, Angptl4, Fabp5, Dbi, Sorbs1, Acsl3, Cyp4a2, Apoa5, Acadl, Cyp4a3, Cpt2, Cyp8b1, Fabp4, Slc27a2, Cyp4a1, Fads2, Acsl1
rno00071:Fatty acid degradation	13	40.62	8.33E-21	7.95E-18	Cpt1b, Acsl3, Cyp4a2, Cpt1c, Acadl, Cyp4a3, Cpt1a, Cpt2, Ehhadh, Acaa1a, Acadm, Cyp4a1, Acsl1
rno01212:Fatty acid metabolism	11	34.375	8.23E-16	7.43E-13	Cpt1b, Cpt2, Ehhadh, Acsl3, Acaa1a, Cpt1c, Acadl, Acadm, Cpt1a, Fads2, Acsl1
rno04920:Adipocytokine signaling pathway	5	15.625	1.18E-4	0.112	Cpt1b, Acsl3, Cpt1c, Cpt1a, Acsl1
rno04146:Peroxisome	5	15.625	1.92E-4	0.1833	Ehhadh, Acsl3, Acaa1a, Slc27a2, Acsl1
rno01100:Metabolic pathways	13	40.625	6.33E-4	0.60323	Acsl3, Cyp4a2, Hmgcs2, Acadl, Cyp4a3, Cyp27a1, Me1, Cyp8b1, Ehhadh, Acaa1a, Acadm, Cyp4a1, Acsl1
rno00280:Valine, leucine and isoleucine degradation	4	12.5	8.67E-4	0.82457	Ehhadh, Acaa1a, Hmgcs2, Acadm
rno04931:Insulin resistance	4	12.5	0.006284	5.83879	Cpt1b, Pdpk1, Slc27a2, Cpt1a
rno04152:AMPK signaling pathway	4	12.5	0.0093345	8.561497	Cpt1b, Cpt1c, Pdpk1, Cpt1a
rno00590:Arachidonic acid metabolism	3	9.375	0.03176	26.510716	Cyp4a2, Cyp4a3, Cyp4a1
rno00830:Retinol metabolism	3	9.375	0.033217	27.559409	Cyp4a2, Cyp4a3, Cyp4a1
rno01130:Biosynthesis of antibiotics	4	12.5	0.0391	31.695763	Ehhadh, Acaa1a, Hmgcs2, Acadm
rno04922:Glucagon signaling pathway	3	9.375	0.046616	36.592474	Cpt1b, Cpt1c, Cpt1a
rno00061:Fatty acid biosynthesis	2	6.25	0.047544	37.179017	Acsl3, Acsl1
rno00120:Primary bile acid biosynthesis	2	6.25	0.054155	41.219565	Cyp8b1, Cyp27a1
rno04750:Inflammatory mediator regulation of TRP channels	3	9.375	0.05981	44.4906	Cyp4a2, Cyp4a3, Cyp4a1
rno01200:Carbon metabolism	3	9.375	0.06541	47.56900	Me1, Ehhadh, Acadm

Table S8: Functional GO annotation on the biomarker genes in co-cluster-1 discovered by the proposed LPHVM algorithm in the case of PPAR signaling pathway data analysis.

**Term**	**Count**	**%**	**P-value**	**FDR**	**Genes**
GO:0008152~metabolic process	28	87.5	7.42E-5	0.055	Cpt1b, Cpt1c, Pdpk1, Hmgcs2, Cpt1a, Cyp27a1, Me1, Plin5, Fabp3, Ehhadh, Acaa1a, Acadm, Angptl4, Fabp5, Dbi, Sorbs1, Acsl3, Cyp4a2, Apoa5, Acadl, Cyp4a3, Cpt2, Cyp8b1, Fabp4, Slc27a2, Cyp4a1, Fads2, Acsl1
GO:0044699~single-organism process	30	93.75	0.00106	0.7931	Plin2, Cpt1b, Aqp7, Cpt1c, Pdpk1, Hmgcs2, Cpt1a, Cyp27a1, Me1, Plin5, Fabp3, Ehhadh, Acaa1a, Acadm, Angptl4, Fabp5, Dbi, Sorbs1, Acsl3, Cyp4a2, Apoa5, Acadl, Cyp4a3, Cpt2, Cyp8b1, Slc27a2, Fabp4, Cyp4a1, Fads2, Acsl1
GO:0051179~localization	16	50.0	0.0190	13.3934	Dbi, Cpt1b, Plin2, Sorbs1, Aqp7, Acsl3, Apoa5, Pdpk1, Cpt1a, Plin5, Cpt2, Fabp3, Fabp4, Slc27a2, Acsl1, Fabp5

Table S9: Partitioning the biomarker genes into upregulated and downregulated biomarker genes for simulated datasets.

**Dataset**	**Biomarker genes**
D_1	G1, G2, G3, G4, G5, G6, G7, G11, G12, G13, G14, G15, G16, G17, G8, G9, G10, G18, G19, G20
D_2	G1, G2, G3, G4, G5, G6, G7, G11, G12, G13, G14, G15, G16, G17, G8, G9, G10, G18, G19, G20

Table S10: Biomarker genes regulatory deses of chemical compounds ranking for simulated datasets (D_1 and D_2).

**Dataset**	**Doses of chemical compounds**	**Percent score**	**Doses of chemical compounds**	**Percent score**
D_1	C8_High	100.00	C7_Middle	98.68
	C7_High	99.89	C10_High	98.61
	C4_Middle	99.79	C6_High	98.43
	C9_High	99.66	C9_Middle	98.26
	C3_High	99.61	C10_Middle	97.98
	C2_High	99.44	C1_High	97.74
	C5_High	99.37	C6_Middle	97.46
	C1_Middle	99.07	C2_Middle	97.46
	C5_Middle	98.98	C8_Middle	97.06
	C4_High	98.72	C3_Middle	97.02
D_2	C7_High	100.00	C1_High	95.95
	C17_High	99.85	C13_High	95.74
	C5_High	99.48	C13_Middle	95.67
	C2_High	99.44	C12_Middle	95.52
	C12_High	98.07	C17_Middle	95.38
	C3_High	97.80	C15_High	95.35
	C2_Middle	97.61	C4_Middle	95.02
	C14_High	97.06	C15_Middle	94.81
	C16_High	97.03	C14_Middle	94.55
	C6_Middle	96.75	C7_Middle	94.48
	C11_High	96.72	C16_Middle	94.44
	C6_High	96.50	C1_Middle	94.34
	C11_Middle	96.12	C3_Middle	94.15
	C4_High	96.09	C5_Middle	92.09

Table S11: Biomarker genes regulatory doses of chemical compounds ranking for glutathione metabolism pathway considering all time points (3hr, 6hr, 9hr and 24hr).

**Doses of chemical compounds**	**Percent score**
nitrofurazone_High_24.hr	100.00
acetaminophen_High_24.hr	99.11
acetaminophen_Middle_24.hr	97.38
methapyrilene_High_24.hr	96.87
nitrofurazone_High_9.hr	96.78
methapyrilene_High_9.hr	95.98
nitrofurazone_Middle_9.hr	95.05
methapyrilene_High_6.hr	94.28
nitrofurazone_High_6.hr	94.25
acetaminophen_Middle_9.hr	93.56
nitrofurazone_Low_6.hr	93.15
nitrofurazone_Low_9.hr	93.02
nitrofurazone_Middle_24.hr	91.56
nitrofurazone_Middle_6.hr	90.60
isoniazid_High_9.hr	90.59
acetaminophen_Low_9.hr	89.82
methapyrilene_Middle_6.hr	88.51
acetaminophen_Low_24.hr	88.21
gentamicin_High_6.hr	87.76
penicillamine_High_6.hr	87.70
penicillamine_High_9.hr	87.51
methapyrilene_Middle_3.hr	87.26
isoniazid_Low_3.hr	87.26
perhexiline_Middle_9.hr	87.04
isoniazid_Middle_3.hr	87.00
methapyrilene_High_3.hr	86.98
isoniazid_Middle_9.hr	86.53
acetaminophen_High_3.hr	86.12
erythromycin.ethylsuccinate_High_9.hr	86.10
erythromycin.ethylsuccinate_Low_6.hr	85.88
erythromycin.ethylsuccinate_Low_9.hr	85.81
nitrofurazone_Middle_3.hr	85.71
acetaminophen_Low_3.hr	85.47
methapyrilene_Low_24.hr	85.42
methapyrilene_Low_3.hr	85.41
nitrofurazone_High_3.hr	85.12

Table S12: Ranking of probabilistic relationships between biomarker genes and their regulatory doses of chemical compounds for glutathione metabolism pathway.

Doses of chemical compounds	Biomarker gene	Ranking score	Doses of chemical compounds	Biomarker gene	Ranking score
acetaminophen_High	Gsta5	100.00	acetaminophen_High	Mgst3	63.11
nitrofurazone_High	Gsta5	96.26	nitrofurazone_Middle	Gstp1	63.02
acetaminophen_Middle	Gsta5	91.69	methapyrilene_High	Oplah	62.55
acetaminophen_High	G6pd	90.85	nitrofurazone_High	Gstm1	62.40
acetaminophen_High	Gpx2	89.67	nitrofurazone_Middle	Gclc	62.27
nitrofurazone_High	G6pd	89.48	nitrofurazone_Middle	Ggt5	62.20
nitrofurazone_High	Gpx2	89.29	nitrofurazone_Middle	Mgst2	62.06
acetaminophen_Middle	Gpx2	86.05	nitrofurazone_High	Gsta4	62.00
acetaminophen_Middle	G6pd	85.91	acetaminophen_Middle	Mgst3	61.95
acetaminophen_High	Gsr	85.19	nitrofurazone_Middle	Gclm	61.90
acetaminophen_High	Gstp1	83.54	methapyrilene_High	Sms	61.86
nitrofurazone_High	Gsr	83.25	nitrofurazone_Middle	Rrm1	61.46
nitrofurazone_High	Gstp1	81.53	acetaminophen_High	Gstm1	61.28
acetaminophen_High	Mgst2	80.46	nitrofurazone_Middle	LOC100912604/Srm	60.82
acetaminophen_High	Gclc	80.38	acetaminophen_High	Gsta4	60.81
methapyrilene_High	Gsta5	80.23	nitrofurazone_High	Hpgds	60.69
acetaminophen_Middle	Gsr	79.71	methapyrilene_High	Gstm7	60.68
acetaminophen_High	Gclm	79.56	acetaminophen_Middle	Gstm1	60.58
methapyrilene_High	Gpx2	79.47	hexachlorobenzene_Low	Gsta5	60.46
nitrofurazone_High	Gclc	78.93	acetaminophen_Middle	Gsta4	60.21
nitrofurazone_High	Mgst2	78.92	acetaminophen_Low	Gsr	59.93
nitrofurazone_High	Gclm	78.20	acetaminophen_Low	Ggt5	59.86
acetaminophen_Middle	Gstp1	78.03	nitrofurazone_Middle	Gss	59.43
methapyrilene_High	G6pd	78.01	acetaminophen_High	Hpgds	59.39
acetaminophen_High	LOC100912604/Srm	75.78	nitrofurazone_Middle	Gsta2/Gsta5	59.33
acetaminophen_Middle	Gclc	75.70	nitrofurazone_Middle	Gstm4	59.23
acetaminophen_Middle	Mgst2	75.67	acetaminophen_Low	Rrm1	59.23
nitrofurazone_High	LOC100912604/Srm	75.03	nitrofurazone_Middle	Odc1	59.16
acetaminophen_Middle	Gclm	75.03	acetaminophen_Middle	Hpgds	58.98
nitrofurazone_Middle	Gpx2	73.70	nitrofurazone_Middle	Oplah	58.79
acetaminophen_High	Gstm4	73.46	methapyrilene_High	Mgst3	58.66
nitrofurazone_High	Gstm4	72.81	acetaminophen_Low	Gstp1	58.36
acetaminophen_High	Gss	72.32	nitrofurazone_Middle	Sms	58.31
acetaminophen_Middle	LOC100912604/Srm	72.17	acetaminophen_Low	Gclc	58.06
nitrofurazone_High	Gss	72.02	hexachlorobenzene_Low	Ggt5	57.90
methapyrilene_High	Gsr	71.48	acetaminophen_Low	Mgst2	57.81
nitrofurazone_Middle	G6pd	71.31	methapyrilene_High	Gstm1	57.79
nitrofurazone_High	Gsta2/Gsta5	70.56	acetaminophen_Low	Gclm	57.78
acetaminophen_High	Gsta2/Gsta5	70.43	methapyrilene_High	Gsta4	57.53
acetaminophen_High	Ggt5	70.21	nitrofurazone_Middle	Gstm7	57.49
nitrofurazone_High	Rrm1	70.09	hexachlorobenzene_Low	Rrm1	57.32
acetaminophen_Middle	Gstm4	70.06	acetaminophen_Low	LOC100912604/Srm	57.23
nitrofurazone_High	Odc1	69.89	acetaminophen_Low	LOC100912604/Srm	57.23
methapyrilene_High	Gstp1	69.83	hexachlorobenzene_Low	Gsr	56.74
acetaminophen_Low	Gpx2	69.74	methapyrilene_High	Hpgds	56.55
acetaminophen_High	Odc1	69.61	acetaminophen_Low	Gsta2/Gsta5	56.47
acetaminophen_Middle	Gss	69.40	acetaminophen_Low	Odc1	56.42
acetaminophen_Middle	Ggt5	69.08	acetaminophen_Low	Gss	56.24
acetaminophen_High	Rrm1	68.98	acetaminophen_Low	Oplah	56.20
nitrofurazone_High:	Oplah	68.93	acetaminophen_Low	Sms	55.85
acetaminophen_High	Oplah	68.48	acetaminophen_Low	Gstm4	55.79
methapyrilene_High	Gclc	68.41	nitrofurazone_Middle	Mgst3	55.54
methapyrilene_High	Mgst2	68.28	acetaminophen_Low	Gstm7	55.28
acetaminophen_Middle	Gsta2/Gsta5	68.13	hexachlorobenzene_Low	Gstp1	55.21
acetaminophen_Middle	Rrm1	68.00	hexachlorobenzene_Low	Gclc	55.15
methapyrilene_High	Gclm	67.91	nitrofurazone_Middle	Gstm1	55.05
nitrofurazone_High	Sms	67.88	hexachlorobenzene_Low	Gclm	54.92
acetaminophen_Middle	Odc1	67.54	hexachlorobenzene_Low	Mgst2	54.88
acetaminophen_High	Sms	67.27	nitrofurazone_Middle	Gsta4	54.87

Doses of chemical compounds	Biomarker gene	Ranking score	Doses of chemical compounds	Biomarker gene	Ranking score
hexachlorobenzene_Low	Gpx2	66.80	hexachlorobenzene_Low	LOC100912604/Srm	54.64
acetaminophen_Low	G6pd	66.72	hexachlorobenzene_Low	Odc1	54.28
acetaminophen_Middle	Oplah	66.66	hexachlorobenzene_Low	Gsta2/Gsta5	54.27
methapyrilene_High	LOC100912604/Srm	66.07	hexachlorobenzene_Low	Oplah	54.14
nitrofurazone_High	Gstm7	66.07	nitrofurazone_Middle	Hpgds	54.08
acetaminophen_Middle	Sms	65.70	hexachlorobenzene_Low	Sms	53.87
methapyrilene_High	Ggt5	65.56	hexachlorobenzene_Low	Gss	53.87
acetaminophen_High	Gstm7	65.19	hexachlorobenzene_Low	Gstm7	53.43
methapyrilene_High	Rrm1	64.68	acetaminophen_Low	Mgst3	53.38
nitrofurazone_Middle	Gsr	64.62	hexachlorobenzene_Low	Gstm4	53.30
acetaminophen_Low	Gsta5	64.56	acetaminophen_Low	Gstm1	53.15
methapyrilene_High	Gstm4	64.25	acetaminophen_Low	Gsta4	53.02
methapyrilene_High	Gss	64.09	acetaminophen_Low	Hpgds	52.36
acetaminophen_Middle	Gstm7	64.04	hexachlorobenzene_Low	Mgst3	51.58
nitrofurazone_High	Mgst3	63.92	hexachlorobenzene_Low	Gstm1	51.49
hexachlorobenzene_Low	G6pd	63.50	hexachlorobenzene_Low	Gsta4	51.39
methapyrilene_High	Gsta2/Gsta5	63.49	hexachlorobenzene_Low	Hpgds	50.81
methapyrilene_High	Odc1	63.14			

Table S13: Ranking of probabilistic relationships between biomarker genes and their regulatory doses of chemical compounds for PPAR signaling pathway.

Chemical compound and dose combination	Biomarker gene	Ranking score	Chemical compound and dose combination	Biomarker gene	Ranking score
WY14643_High	Ehhadh	100.00	WY14643_Low	Aqp7	61.88
WY14643_High	Cyp4a1	97.29	gemfibrozil_Middle	Me1	61.63
WY14643_Middle	Ehhadh	95.32	benzbromarone_High	Cyp4a2	61.57
WY14643_Middle	Cyp4a1	93.17	clofibrate_High	Fads2	61.56
WY14643_High	Acaa1a	92.41	clofibrate_High	Acsl3	61.52
clofibrate_High	Ehhadh	88.93	benzbromarone_High	Fads2	61.22
WY14643_Middle	Acaa1a	88.47	aspirin_Low	Cpt1c	61.19
clofibrate_High	Cyp4a1	87.34	benzbromarone_Middle	Cyp8b1	61.11
benzbromarone_High	Ehhadh	87.04	benzbromarone_High	Acsl3	61.02
WY14643_High	Cyp4a3	86.68	clofibrate_High	Fabp3	61.01
WY14643_Low	Ehhadh	86.65	gemfibrozil_High	Cpt1c	61.00
WY14643_High	Plin5	85.99	WY14643_High	Plin2	60.86
benzbromarone_High	Cyp4a1	85.67	benzbromarone_Middle	Fabp5	60.72
WY14643_Low	Cyp4a1	85.17	WY14643_Low	Cyp4a2	60.71
WY14643_High	Cpt2	84.46	clofibrate_High	Sorbs1	60.60
WY14643_High	Cpt1b	84.45	benzbromarone_High	Fabp3	60.58
WY14643_High	Angptl4	83.99	aspirin_High	Sorbs1	60.51
aspirin_High	Ehhadh	83.60	WY14643_Low	Fads2	60.30
WY14643_Middle	Cyp4a3	83.54	WY14643_Low	Acsl3	60.21
aspirin_High	Cyp4a1	83.10	aspirin_High	Fabp4	60.19
WY14643_Middle	Plin5	83.00	aspirin_High	Acadm	60.16
clofibrate_High	Acaa1a	82.91	aspirin_Middle	Aqp7	60.06
WY14643_Middle	Angptl4	81.62	WY14643_High	Fabp4	59.98
benzbromarone_High	Acaa1a	81.31	benzbromarone_High	Sorbs1	59.98
WY14643_Middle	Cpt1b	81.18	gemfibrozil_Middle	Cpt1c	59.94
WY14643_Middle	Cpt2	81.16	WY14643_High	Hmgcs2	59.93
WY14643_Low	Acaa1a	80.84	aspirin_High	Slc27a2	59.92
WY14643_High	LOC100910385	80.26	WY14643_Middle	Plin2	59.90
clofibrate_High	Cyp4a3	78.82	WY14643_High	Cyp27a1	59.88
aspirin_High	Acaa1a	78.81	benzbromarone_Middle	Cpt1b	59.86
aspirin_Middle	Ehhadh	78.49	aspirin_Middle	Fads2	59.84
clofibrate_High	Plin5	78.45	clofibrate_High	Acadm	59.76
aspirin_Middle	Cyp4a1	78.16	aspirin_Middle	Cyp4a2	59.76
WY14643_High	Fabp5	77.70	WY14643_Low	Fabp3	59.73
clofibrate_High	Angptl4	77.67	benzbromarone_Middle	Cpt2	59.68

Chemical compound and dose combination	Biomarker gene	Ranking score	Chemical compound and dose combination	Biomarker gene	Ranking score
benzbromarone_High	Cyp4a3	77.54	WY14643_Middle	Fabp4	59.63
WY14643_High	LOC100365047	77.44	benzbromarone_Middle	LOC100365047	59.60
WY14643_Middle	LOC100910385	77.29	WY14643_High	Acadl	59.56
benzbromarone_High	Plin5	77.23	WY14643_High	Apoa5	59.55
aspirin_Low	Ehhadh	77.05	clofibrate_High	Slc27a2	59.46
WY14643_Low	Cyp4a3	76.93	WY14643_Low	Sorbs1	59.27
aspirin_Low	Cyp4a1	76.74	benzbromarone_High	Acadm	59.24
benzbromarone_High	Angptl4	76.69	WY14643_High	Pdpk1	59.18
aspirin_High	Angptl4	76.62	WY14643_Middle	Hmgcs2	59.13
WY14643_Low	Plin5	76.58	WY14643_Middle	Cyp27a1	59.08
clofibrate_High	Cpt1b	76.40	aspirin_Low	Aqp7	59.04
clofibrate_High	Cpt2	76.35	benzbromarone_High	Slc27a2	58.95
aspirin_High	Cyp4a3	76.20	aspirin_Middle	Acsl3	58.92
aspirin_High	Plin5	76.14	aspirin_Low	Fads2	58.84
WY14643_Low	Angptl4	75.90	WY14643_Middle	Apoa5	58.81
WY14643_Middle	Fabp5	75.71	aspirin_Middle	Fabp3	58.79
WY14643_Middle	LOC100365047	75.29	aspirin_Low	Cyp4a2	58.75
gemfibrozil_High	Ehhadh	75.15	WY14643_Middle	Acadl	58.63
gemfibrozil_Middle	Ehhadh	75.14	benzbromarone_Middle	Cpt1a	58.62
gemfibrozil_High	Cyp4a1	75.08	aspirin_High	Plin2	58.62
benzbromarone_High	Cpt1b	75.07	gemfibrozil_High	Fads2	58.60
benzbromarone_High	Cpt2	75.01	WY14643_Middle	Pdpk1	58.57
gemfibrozil_Middle	Cyp4a1	74.89	gemfibrozil_High	Aqp7	58.51
WY14643_Low	Cpt1b	74.54	benzbromarone_Middle	Me1	58.51
WY14643_Low	Cpt2	74.49	WY14643_Low	Acadm	58.48
aspirin_Middle	Acaa1a	74.11	gemfibrozil_High	Cyp4a2	58.40
WY14643_High	Cyp8b1	73.70	aspirin_High	Hmgcs2	58.32
aspirin_High	Cpt1b	73.40	aspirin_High	Pdpk1	58.32
aspirin_High	Cpt2	73.27	aspirin_High	Cyp27a1	58.25
WY14643_High	Cpt1a	72.92	WY14643_Low	Slc27a2	58.18
clofibrate_High	LOC100910385	72.87	aspirin_High	Apoa5	58.17
aspirin_Low	Acaa1a	72.77	clofibrate_High	Fabp4	58.00
WY14643_Middle	Cyp8b1	72.44	aspirin_Low	Acsl3	57.92
aspirin_Middle	Angptl4	72.42	benzbromarone_High	Fabp4	57.83
clofibrate_High	Fabp5	72.21	aspirin_Low	Fabp3	57.80
aspirin_Middle	Cyp4a3	71.82	gemfibrozil_Middle	Aqp7	57.76
aspirin_Middle	Plin5	71.81	clofibrate_High	Plin2	57.71
aspirin_High	Fabp5	71.67	benzbromarone_Middle	Cpt1c	57.67
clofibrate_High	LOC100365047	71.66	benzbromarone_Middle	LOC100910385	57.64
benzbromarone_High	LOC100910385	71.66	gemfibrozil_Middle	Fads2	57.63
benzbromarone_High	Fabp5	71.39	gemfibrozil_Middle	Cyp4a2	57.52
WY14643_Middle	Cpt1a	71.34	gemfibrozil_High	Acsl3	57.51
gemfibrozil_High	Acaa1a	71.18	gemfibrozil_High	Fabp3	57.46
aspirin_Low	Angptl4	71.16	aspirin_High	Acadl	57.41
WY14643_Low	LOC100910385	71.11	benzbromarone_High	Plin2	57.31
gemfibrozil_Middle	Acaa1a	71.01	aspirin_Middle	Sorbs1	57.28
benzbromarone_High	LOC100365047	70.78	aspirin_Middle	Fabp4	57.25
aspirin_High	LOC100365047	70.76	clofibrate_High	Hmgcs2	57.11
WY14643_Low	Fabp5	70.60	clofibrate_High	Cyp27a1	57.05
aspirin_High	Cyp8b1	70.55	aspirin_Middle	Acadm	57.00
aspirin_Low	Cyp4a3	70.54	WY14643_Low	Fabp4	56.87
WY14643_High	Me1	70.54	clofibrate_High	Apoa5	56.85
aspirin_Low	Plin5	70.53	aspirin_Middle	Slc27a2	56.78
aspirin_High	LOC100910385	70.31	benzbromarone_High	Hmgcs2	56.77
gemfibrozil_High	Angptl4	70.25	clofibrate_High	Pdpk1	56.73
WY14643_Low	LOC100365047	70.04	benzbromarone_High	Cyp27a1	56.70
clofibrate_High	Cyp8b1	69.69	gemfibrozil_Middle	Acsl3	56.69
gemfibrozil_Middle	Angptl4	69.57	gemfibrozil_Middle	Fabp3	56.59
gemfibrozil_High	Plin5	69.35	benzbromarone_High	Apoa5	56.53
WY14643_Middle	Me1	69.33	WY14643_Low	Plin2	56.50
gemfibrozil_High	Cyp4a3	69.29	clofibrate_High	Acadl	56.50
benzbromarone_High	Cyp8b1	69.16	WY14643_High	Dbi	56.47
aspirin_Middle	Cpt1b	69.13	benzbromarone_High	Pdpk1	56.46

**Chemical compound and dose combination**	**Biomarker gene**	**Ranking score**	**Chemical compound and dose combination**	**Biomarker gene**	**Ranking score**
aspirin_Middle	Cpt2	68.99	aspirin_Low	Fabp4	56.31
gemfibrozil_Middle	Plin5	68.90	aspirin_Low	Sorbs1	56.29
gemfibrozil_Middle	Cyp4a3	68.89	gemfibrozil_High	Fabp4	56.25
aspirin_High	Cpt1a	68.46	benzbromarone_High	Acadl	56.11
clofibrate_High	Cpt1a	68.33	aspirin_Low	Acadm	56.03
WY14643_Low	Cyp8b1	68.22	WY14643_Middle	Dbi	55.97
aspirin_Low	Cpt1b	67.88	aspirin_High	Dbi	55.96
aspirin_Middle	Fabp5	67.78	WY14643_Low	Hmgcs2	55.93
aspirin_Low	Cpt2	67.75	WY14643_Low	Cyp27a1	55.87
benzbromarone_High	Cpt1a	67.67	aspirin_Low	Slc27a2	55.82
aspirin_High	Me1	67.54	gemfibrozil_High	Sorbs1	55.73
WY14643_High	Aqp7	67.46	WY14643_Low	Apoa5	55.68
benzbromarone_Middle	Cyp4a1	66.97	aspirin_Middle	Plin2	55.61
aspirin_Middle	Cyp8b1	66.90	WY14643_Low	Pdpk1	55.59
aspirin_Middle	LOC100365047	66.88	gemfibrozil_High	Acadm	55.59
WY14643_Low	Cpt1a	66.84	aspirin_Middle	Pdpk1	55.41
clofibrate_High	Me1	66.71	gemfibrozil_High	Slc27a2	55.39
aspirin_Low	Fabp5	66.61	aspirin_Middle	Hmgcs2	55.37
gemfibrozil_High	Cpt1b	66.58	WY14643_Low	Acadl	55.32
gemfibrozil_High	Cpt2	66.43	aspirin_Middle	Cyp27a1	55.29
WY14643_High	Cpt1c	66.42	benzbromarone_Middle	Fads2	55.24
benzbromarone_Middle	Ehhadh	66.35	aspirin_Middle	Apoa5	55.23
gemfibrozil_Middle	Cpt1b	66.28	gemfibrozil_Middle	Fabp4	55.19
aspirin_Middle	LOC100910385	66.25	gemfibrozil_Middle	Sorbs1	55.07
benzbromarone_High	Me1	66.19	gemfibrozil_Middle	Acadm	54.84
gemfibrozil_Middle	Cpt2	66.15	benzbromarone_Middle	Cyp4a2	54.77
WY14643_Middle	Aqp7	66.02	aspirin_Low	Plin2	54.67
gemfibrozil_High	Fabp5	65.85	gemfibrozil_Middle	Slc27a2	54.63
aspirin_Low	Cyp8b1	65.77	aspirin_Low	Pdpk1	54.49
WY14643_Middle	Cpt1c	65.75	aspirin_Middle	Acadl	54.47
aspirin_Low	LOC100365047	65.72	aspirin_Low	Hmgcs2	54.44
aspirin_High	Cpt1c	65.49	aspirin_Low	Cyp27a1	54.37
WY14643_High	Cyp4a2	65.37	benzbromarone_Middle	Aqp7	54.35
gemfibrozil_High	Cyp8b1	65.33	gemfibrozil_High	Plin2	54.35
WY14643_Low	Me1	65.29	gemfibrozil_High	Pdpk1	54.32
WY14643_High	Acsl3	65.18	aspirin_Low	Apoa5	54.31
gemfibrozil_Middle	Fabp5	65.14	clofibrate_High	Dbi	54.28
aspirin_Low	LOC100910385	65.07	gemfibrozil_High	Hmgcs2	54.19
gemfibrozil_High	LOC100365047	64.89	gemfibrozil_High	Cyp27a1	54.11
WY14643_High	Sorbs1	64.87	gemfibrozil_High	Apoa5	54.08
aspirin_Middle	Cpt1a	64.83	benzbromarone_High	Dbi	54.05
benzbromarone_Middle	Angptl4	64.48	benzbromarone_Middle	Fabp3	53.87
WY14643_High	Fads2	64.46	benzbromarone_Middle	Acsl3	53.71
gemfibrozil_Middle	Cyp8b1	64.38	aspirin_Low	Acadl	53.55
WY14643_Middle	Cyp4a2	64.35	gemfibrozil_Middle	Plin2	53.52
WY14643_High	Fabp3	64.33	benzbromarone_Middle	Fabp4	53.52
gemfibrozil_Middle	LOC100365047	64.25	gemfibrozil_Middle	Pdpk1	53.38
aspirin_Middle	Me1	64.04	gemfibrozil_Middle	Hmgcs2	53.31
WY14643_Middle	Acsl3	64.00	gemfibrozil_Middle	Cyp27a1	53.24
gemfibrozil_High	LOC100910385	63.89	gemfibrozil_High	Acadl	53.24
aspirin_Low	Cpt1a	63.72	WY14643_Low	Dbi	53.19
clofibrate_High	Cpt1c	63.69	aspirin_Middle	Dbi	53.19
WY14643_Middle	Fads2	63.68	gemfibrozil_Middle	Apoa5	53.19
gemfibrozil_Middle	LOC100910385	63.54	aspirin_High	Acsl1	53.00
WY14643_High	Acadm	63.52	gemfibrozil_Middle	Acadl	52.43
benzbromarone_Middle	Acaa1a	63.44	aspirin_Low	Dbi	52.31
aspirin_High	Aqp7	63.42	gemfibrozil_High	Dbi	52.18
benzbromarone_High	Cpt1c	63.39	WY14643_High	Acsl1	51.79
WY14643_Middle	Sorbs1	63.37	benzbromarone_Middle	Acadm	51.78
WY14643_Middle	Fabp3	63.32	WY14643_Middle	Acsl1	51.74
clofibrate_High	Aqp7	63.25	benzbromarone_Middle	Slc27a2	51.64
gemfibrozil_High	Cpt1a	63.14	benzbromarone_Middle	Sorbs1	51.62
WY14643_High	Slc27a2	63.13	benzbromarone_Middle	Pdpk1	51.35

Chemical compound and dose combination	Biomarker gene	Ranking score	Chemical compound and dose combination	Biomarker gene	Ranking score
aspirin_High	Fads2	63.02	gemfibrozil_Middle	Dbi	51.25
aspirin_High	Cyp4a2	62.99	benzbromarone_Middle	Hmgcs2	51.00
aspirin_Low	Me1	62.96	benzbromarone_Middle	Apoa5	50.96
benzbromarone_Middle	Plin5	62.87	benzbromarone_Middle	Plin2	50.95
benzbromarone_High	Aqp7	62.65	benzbromarone_Middle	Cyp27a1	50.91
benzbromarone_Middle	Cyp4a3	62.61	clofibrate_High	Acsl1	50.56
gemfibrozil_High	Me1	62.54	benzbromarone_High	Acsl1	50.51
WY14643_Low	Cpt1c	62.41	aspirin_Middle	Acsl1	50.47
gemfibrozil_Middle	Cpt1a	62.34	benzbromarone_Middle	Acadl	49.93
WY14643_Middle	Acadm	62.27	gemfibrozil_High	Acsl1	49.71
aspirin_Middle	Cpt1c	62.22	aspirin_Low	Acsl1	49.65
aspirin_High	Acsl3	62.15	WY14643_Low	Acsl1	49.60
clofibrate_High	Cyp4a2	62.00	benzbromarone_Middle	Dbi	49.43
aspirin_High	Fabp3	61.97	gemfibrozil_Middle	Acsl1	48.68
WY14643_Middle	Slc27a2	61.92	benzbromarone_Middle	Acsl1	47.61

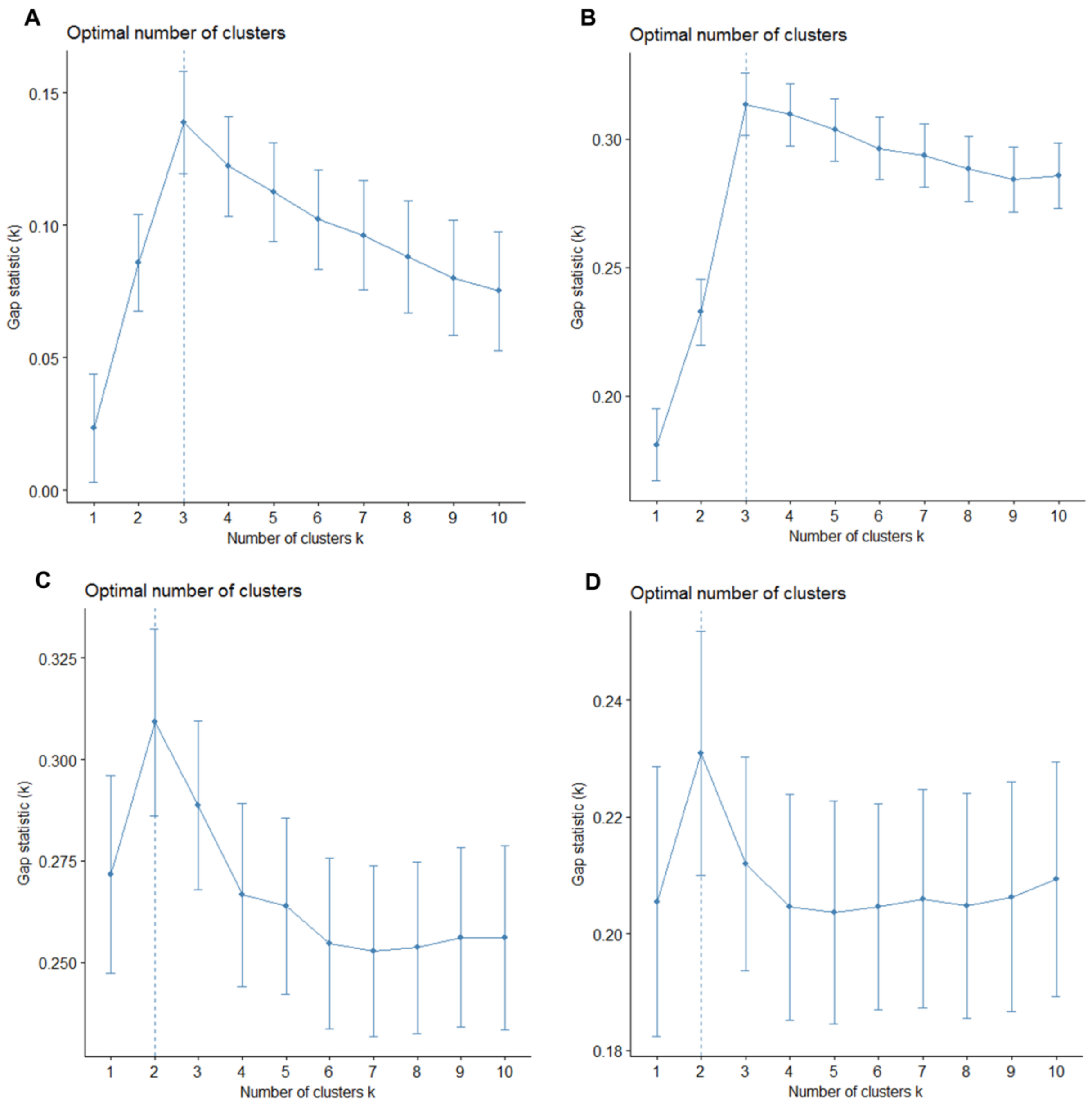

Figure S1: Gap statistic versus number of hidden co-cluster (k) graph for predicting optimal number of hidden co-clusters/clusters. **(A)** For D_1 dataset. **(B)** For D_2 dataset. **(C)** For glutathione metabolism pathway dataset. **(D)** For PPAR signaling pathway dataset.

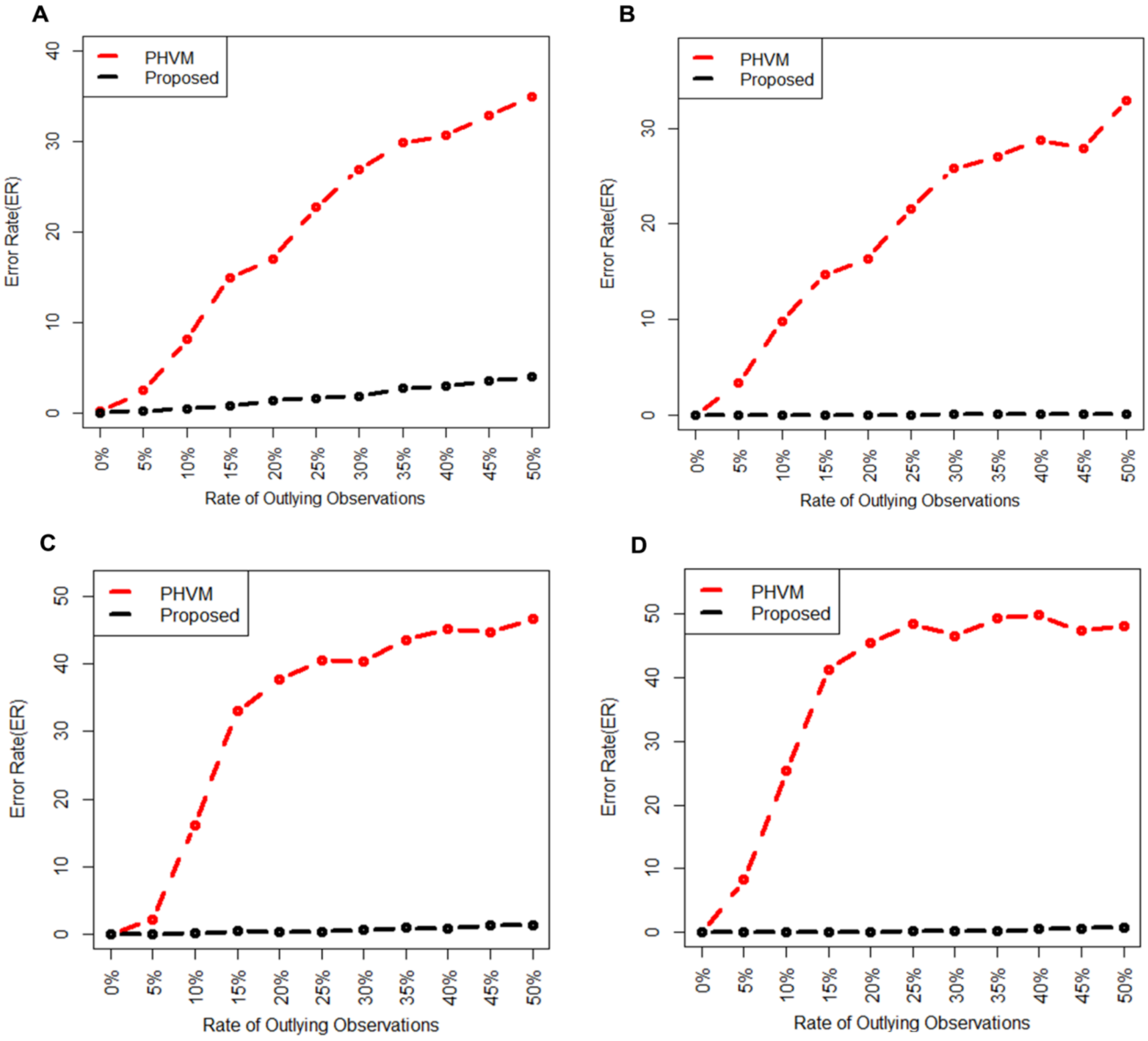

Figure S2: Average genes and doses of chemical compounds clustering ER are plotted against the rate of outliers, when each of the datasets is simulated 100 times and outliers in the dataset are introduced in the dataset by THCM. **(A)** Represents gene clustering ER for D_1 dataset. **(B)** Represents DCCs clustering ER for D_1 dataset. **(C)** Represents gene clustering ER for D_2 dataset. **(D)** DCCs clustering ER for D_2 dataset.

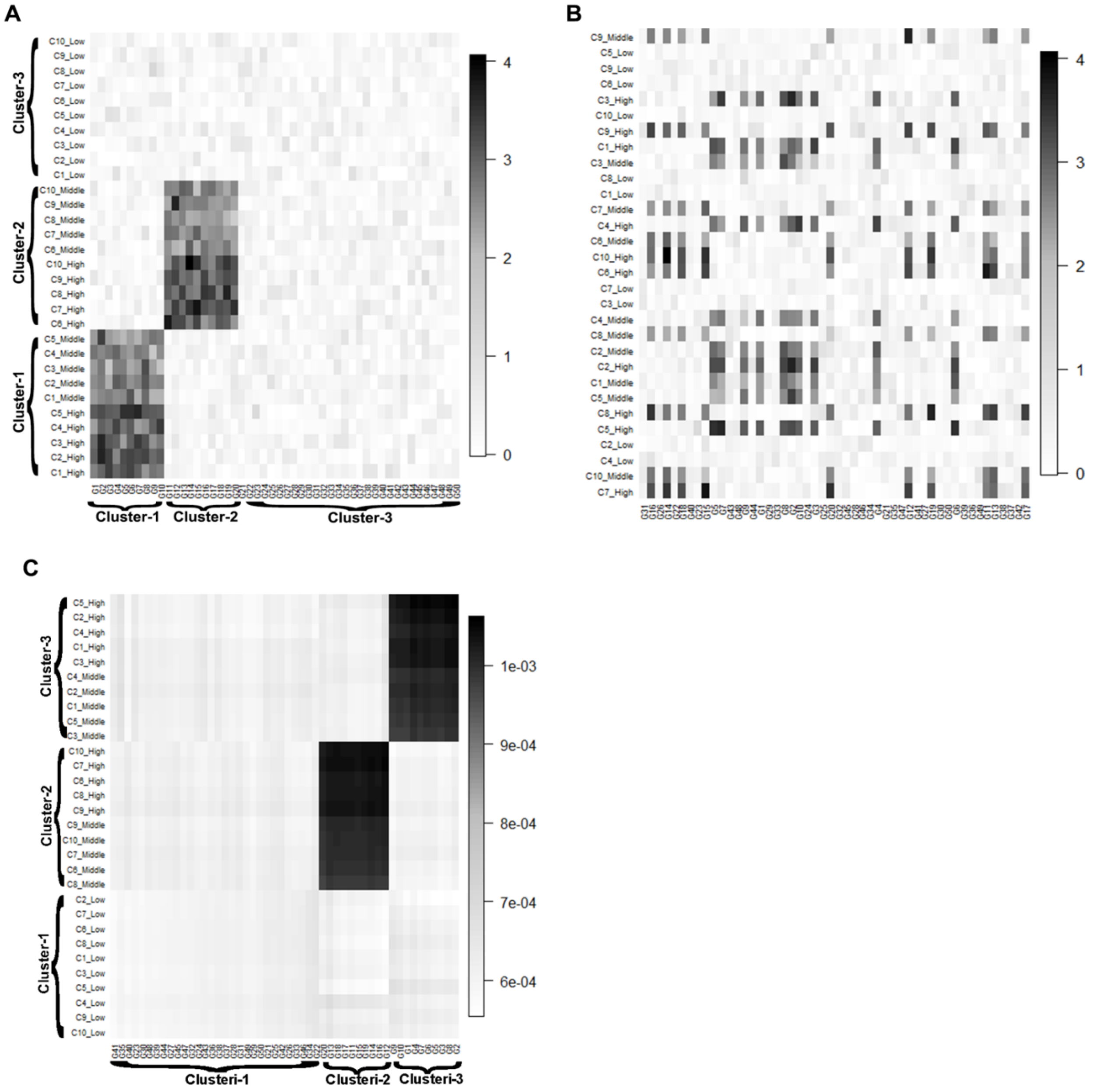

Figure S3: View of simulated and proposed method reconstructed data structure for the dataset D_1. **(A)** Represents the original structure of the simulated data. **(B)** Represents the data structure when gene (row entity) and doses of chemical compounds (column entity) randomly allocated. **(C)** Represents the proposed method reconstructed data structure.

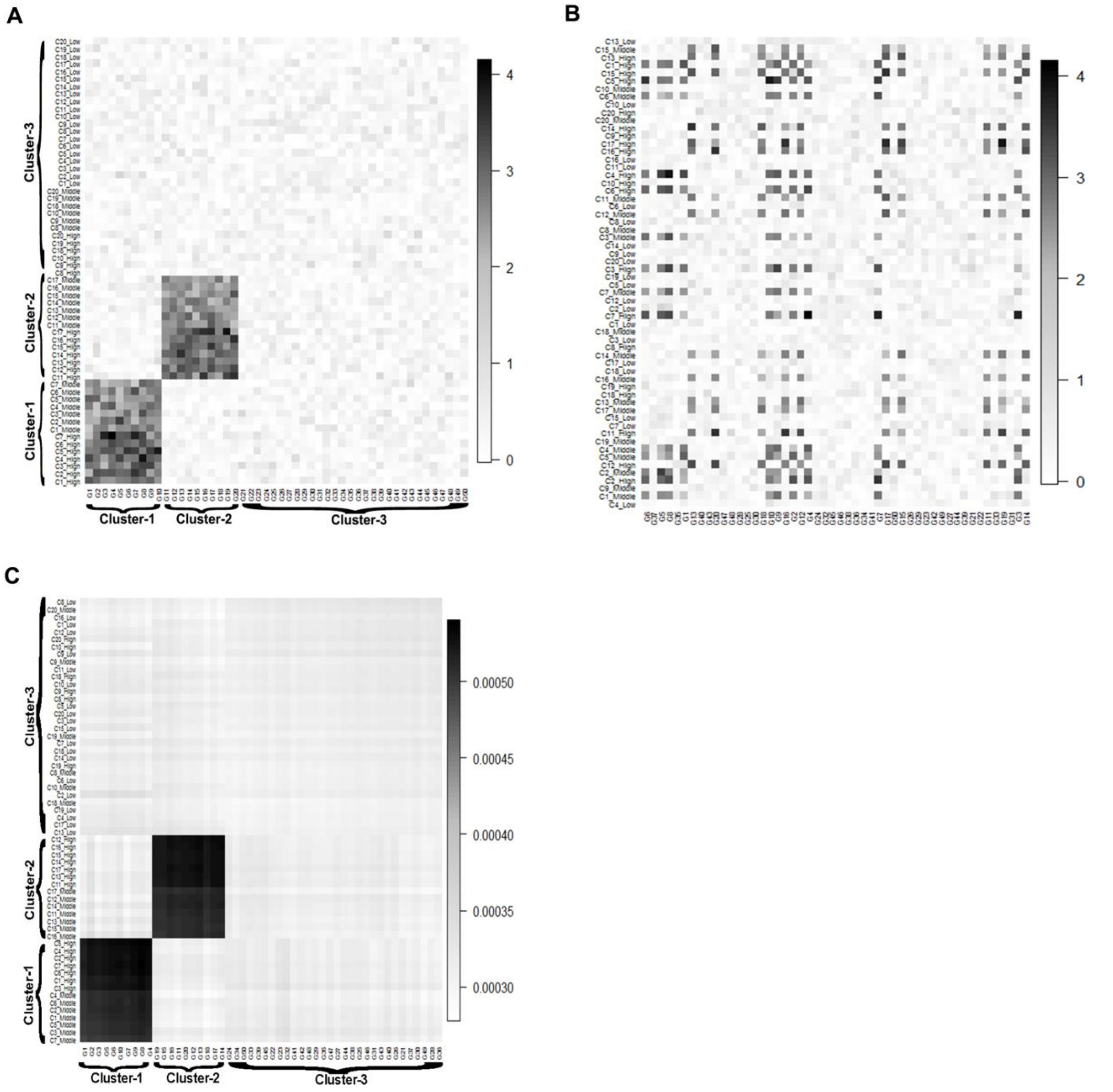

Figure S4: View of simulated and proposed method reconstructed data structure for the dataset D_2. **(A)** Represents the original structure of the simulated data. **(B)** Represents the data structure when gene (row entity) and doses of chemical compounds (column entity) are randomly allocated. **(C)** Represents the proposed method reconstructed data structure.

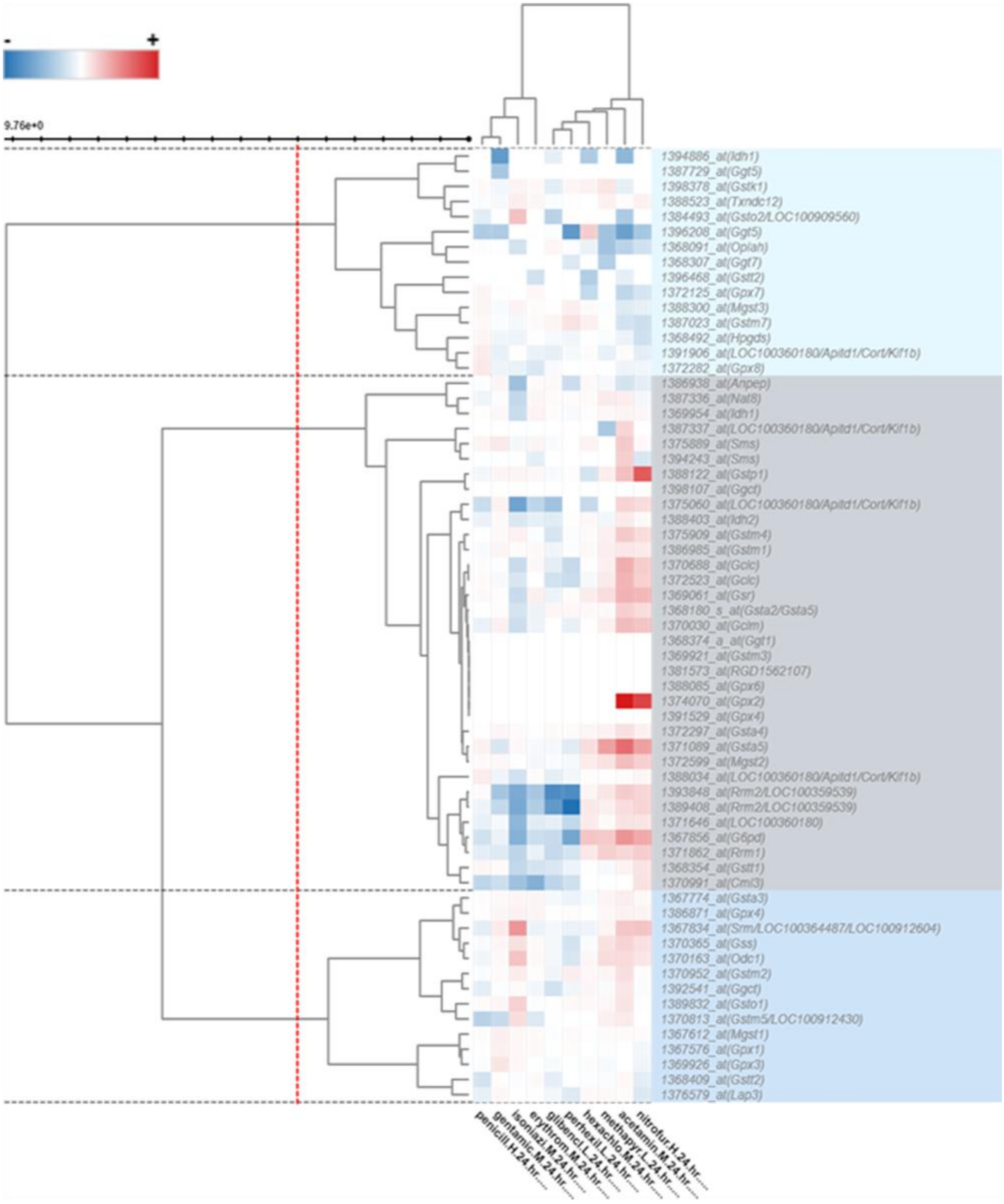

Figure S5: Hirarachical clustering (heatmap) obtained from online toxicogenomic data analysis tool Toxygates for glutathione metabolism pathway genes and some selected compounds (acetaminohen, erythromycin, hexachlorobenzene, isoniazid, gentamicin, glibenclamide, methapyrilene, nitrofurazone, penicillamine and perhexilline) along with dose levels (low, middle, high) at 24 hour time points.

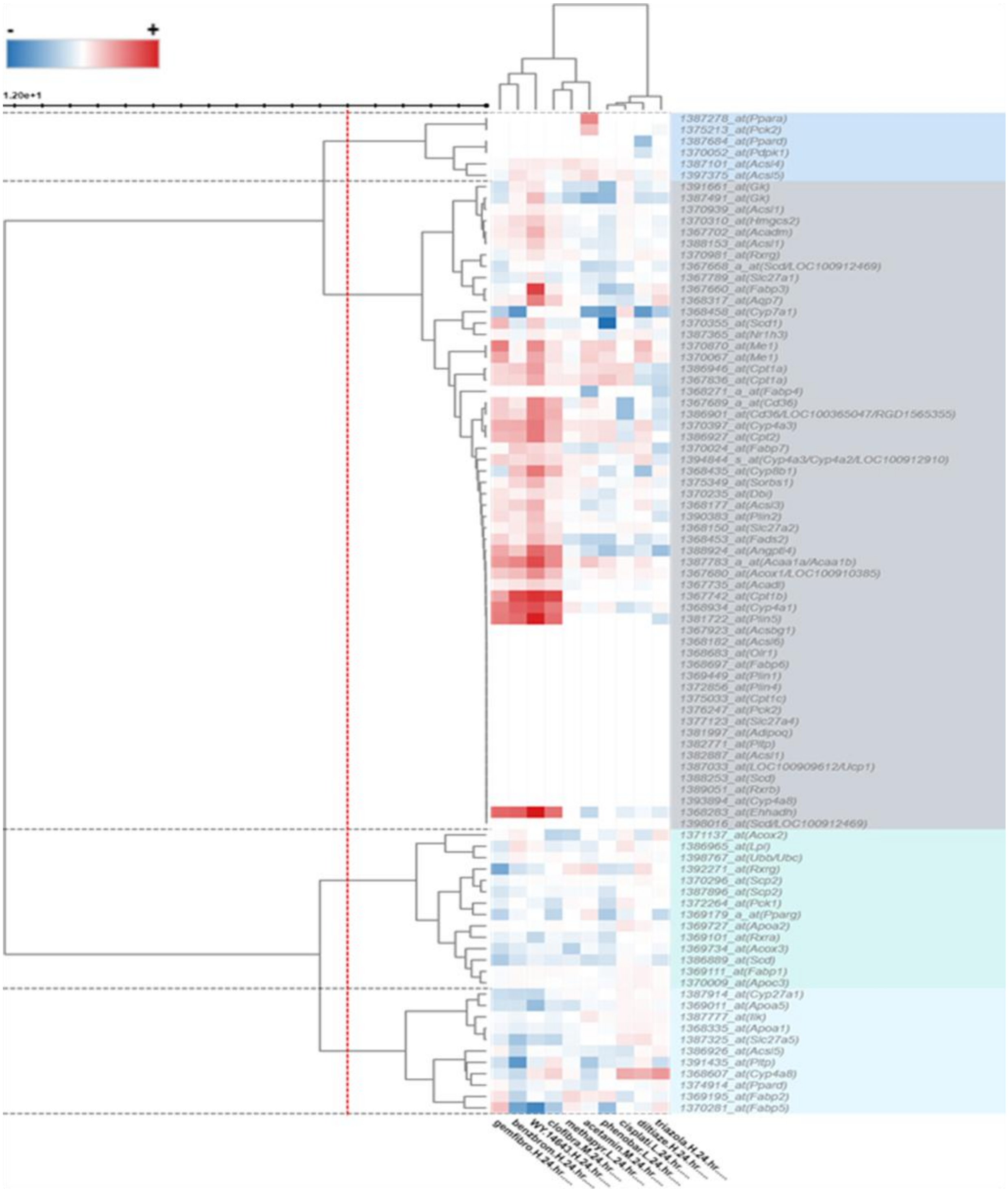

Figure S6: Hirarachical clustering (heatmap) obtained from online toxicogenomic data analysis tool Toxygates for PPAR signaling pathway genes and some selected compounds (acetaminophen, benzbromarone, cisplatin, clofibrate, diltiazem, gemfibrozil, methapyrilene, phenobarbital, triazolam and WY.14643) along with dose levels (low, middle, high) at 24 hour time points.

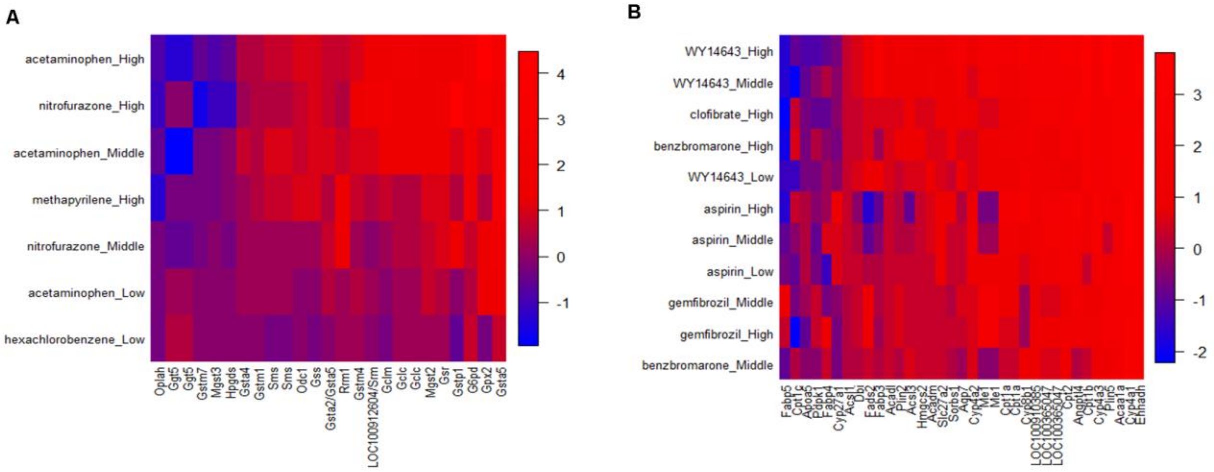

Figure S7: Upregulated and downregulated biomarker genes corresponding to their regulatory doses of chemical compounds at 24 hour time points. **(A)** For glutathione metabolism pathway dataset. **(B)** For PPAR signaling pathway dataset.
